# Association of a Comprehensive Smoking Cessation Program With Smoking Abstinence Among Patients With Cancer

**DOI:** 10.1001/jamanetworkopen.2019.12251

**Published:** 2019-09-27

**Authors:** Paul M. Cinciripini, Maher Karam-Hage, George Kypriotakis, Jason D. Robinson, Vance Rabius, Diane Beneventi, Jennifer A. Minnix, Janice A. Blalock

**Affiliations:** 1 Department of Behavioral Science, The University of Texas MD Anderson Cancer Center, Houston

## Abstract

**Importance:**

Patients with cancer who smoke after diagnosis risk experiencing reductions in treatment effectiveness, survival rates, and quality of life, and increases in complications, cancer recurrence, and second primary cancers. Smoking cessation can significantly affect these outcomes, but to date comprehensive treatment is not widely implemented in the oncologic setting.

**Objectives:**

To describe a potential model tobacco treatment program (TTP) implemented in a cancer setting, report on its long-term outcomes, and highlight its importance to quality patient care.

**Design, Setting, and Participants:**

A prospective cohort of smokers was treated in the TTP at a comprehensive cancer center from January 1, 2006, to August 31, 2015. Data analysis was performed from November 2017 to December 2018. Participants included 3245 patients (2343 with current cancer; 309 with previous cancer; 593 with no cancer history) drawn from a population of 5061 smokers referred for treatment in the TTP. Reasons for exclusion included follow-up for a noncancerous disease, no medical consultation, smoked less than 1 cigarette per day; or died before the 9-month follow-up.

**Exposures:**

Treatment consisted of an in-person medical consultation, 6 to 8 in-person and telephone follow-up counseling sessions, and 10 to 12 weeks of pharmacotherapy.

**Main Outcomes and Measures:**

Primary outcome was 9-month 7-day point-prevalence abstinence evaluated using time-specific (3-, 6-, and 9-month follow-ups) and longitudinal covariate-adjusted and unadjusted regression models with multiple imputation, intention-to-treat, and respondent-only approaches to missing data. The Fagerström Test for Cigarette Dependence was used as a measure of dependence (possible range, 0-10; higher numbers indicate greater dependence).

**Results:**

Of the 3245 smokers, 1588 (48.9%) were men, 322 (9.9%) were of black race/ethnicity, 172 (5.3%) were of Hispanic race/ethnicity, and 2498 (76.0%) were of white race/ethnicity. Mean (SD) age was 54 (11.4) years; Fagerström Test for Cigarette Dependence score, 4.41 (2.2), number of cigarettes smoked per day, 17.1 (10.7); years smoked, 33 (13.2); and 1393 patients (42.9%) had at least 1 psychiatric comorbidity. Overall self-reported abstinence was 45.1% at 3 months, 45.8% at 6 months, and 43.7% at 9 months in the multiply imputed sample. Results across all models were consistent, suggesting that, in comparison with smokers with no cancer history, abstinence rates within this TTP program did not differ appreciably whether smokers had current cancer, were a cancer survivor, or had smoking-related cancers, with the exception of patients with head and neck cancer; the rates were higher at 9 months (relative risk, 1.31; 95% CI, 1.11-1.56; *P* = .001) and in longitudinal models (relative risk, 1.24; 95% CI, 1.08-1.42; *P* = .002).

**Conclusions and Relevance:**

In this study, mean smoking abstinence rates did not differ significantly between patients with cancer and those without cancer. These findings suggest that providing comprehensive tobacco treatment in the oncologic setting can result in sustained high abstinence rates for all patients with cancer and survivors and should be included as standard of care to ensure the best possible cancer treatment outcomes.

## Key Points

**Question:**

Are there differences in abstinence rates between patients with and without cancer after treatment in a comprehensive tobacco cessation program delivered in an oncologic setting?

**Findings:**

In this cohort study of 3245 smokers in a tobacco treatment program, mean smoking abstinence rates were 45.1% at the 3-month follow-up, 45.8% at the 6-month follow-up, and 43.7% at the 9-month follow-up; rates did not differ between patients with and without cancer. Patients with head and neck cancer were among those with the highest abstinence rates.

**Meaning:**

When exposed to a comprehensive tobacco treatment program, smokers with and without cancer showed sustained high quit rates and did not differ from each other, suggesting that comprehensive treatment in an oncologic setting may be successful.

## Teaser

This cohort analysis examines the use of a tobacco treatment program for patients with cancer who continue to smoke after diagnosis.

## Introduction

The 2014 Surgeon General’s Report^1^ concluded that a causal relationship exists between smoking at diagnosis of cancer and both all-cause and cancer-specific mortality, as well as increased risk of disease progression and tobacco-related second primary cancers. Moreover, evidence suggests that continued smoking is associated with increases in the risk of cancer recurrence, poor treatment response, and treatment-related toxic effects.^1^ Smoking cessation at the time of diagnosis has been reported to reduce the risk of dying by 30% to 40%,^1,2^ improve physiologic and psychological functioning,^3,4,5^ and have benefits that equal or exceed those of the best cancer treatments available.^6^

Despite the consensus on the importance of addressing tobacco use in the oncologic setting,^6,7^ many cancer centers^8^ and oncology practices have not fully implemented recommended tobacco assessment and evidence-based treatment practices,^6,7,8,9,10^ including the relatively straightforward Public Health Service guidelines for smoking cessation.^2,6,10,11,12^ Although most patients with cancer are receptive to cessation treatment,^13,14^ oncologists have reported feeling inadequately prepared to deliver interventions,^15,16^ and some believe patients are resistant to treatment, unmotivated to quit, or that quitting smoking is a less immediate concern when beginning cancer treatment.^15,16,17^

In our view, relying on cancer-treating clinicians to deliver the type of specialized smoking cessation treatment called for in the oncologic setting may be unrealistic given the demands on their time, the need for training, and the required motivation to address tobacco use while managing the treatment of a complex disease. While a 2013 review questioned the advantage of specific smoking cessation approaches over usual care in oncologic settings,^18^ that review did not include studies involving a comprehensive and specialist-centered approach to cessation, as is advocated herein. We believe that dedicated tobacco treatment clinicians with a broad understanding of addiction and mental health offer the best means to deliver high-quality and effective smoking cessation treatment to patients with cancer. Integrating tobacco treatment with psychological and medical services in the oncologic setting with the use of 1 or more modalities (eg, face-to-face, telephone, telehealth) can provide focused and seamless patient care while reducing strain on oncology clinicians who face a multitude of demands and requirements to provide a broad spectrum of clinical care.^11,13^ The need for enhancing tobacco treatment in oncology settings has been recognized by leaders in the field,^2,6,8,11,19^ prompting the National Cancer Institute to provide supplemental funding in 2017 and 2018 for 42 cancer centers to develop their tobacco cessation capacity.^20^

This cohort study evaluated the outcome of the tobacco treatment program (TTP) at The University of Texas MD Anderson Cancer Center in Houston. The program began modestly in 2006 with clinician referral, later incorporating automated referral using electronic health records. The program currently treats nearly 1200 new patients and conducts more than 11 000 patient visits per year. The TTP is comprehensive, consisting of individualized smoking cessation counseling, over-the-counter and prescription pharmacotherapy, and the integrated assessment and treatment of mental health conditions and other psychosocial concerns. Herein, we provide program abstinence data and evaluate differences between patients with and without cancer and between cancer sites. Our working hypothesis is that comprehensive care can result in sustained high quit-rates for all patients with cancer, similar to those observed among individuals without cancer who are treated within the same program.

## Methods

The results of this study are reported following the Strengthening the Reporting of Observational Studies in Epidemiology (STROBE) checklist for cohort studies.^21^ This research was approved by the MD Anderson Institutional Review Board, Houston, Texas, as a database protocol with waiver of informed consent.

### Participants

The sample consisted of 3245 patients with and without cancer referred to the MD Anderson Cancer Center TTP between January 1, 2006, and August 31, 2014, and who completed a 9-month follow-up by August 31, 2015. Data analysis was performed from November 2017 to December 2018. The TTP provides counseling and medication free of charge and is available to all current smokers receiving care by an MD Anderson Cancer Center physician as well as patients’ family members and MD Anderson Cancer Center employees. Initially, patients were referred by a clinician or self-referred, including those who self-identified as smokers on a questionnaire administered electronically in some, but not all, clinics at the initial oncologic visit. After July 2013, the questionnaire was administered electronically in all clinics through the electronic health records and referrals were automatically routed to our program staff. However, clinicians could refer directly to the program.

**Figure 1.**
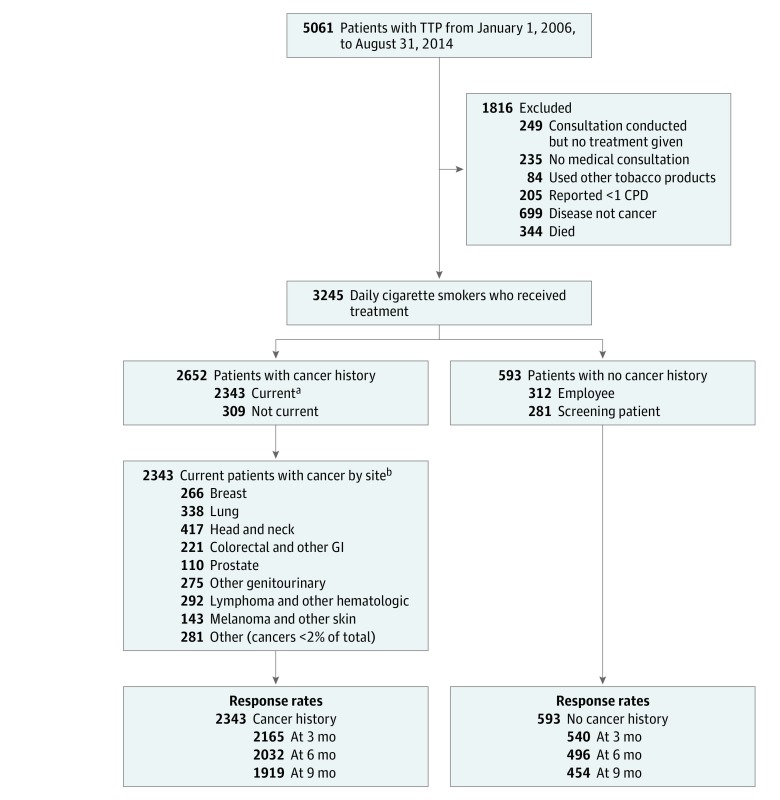
Flow Diagram for Patient Ascertainment CPD indicates cigarettes per day; GI, gastrointestinal; and TTP, tobacco treatment program. ^a^Current cancers were analyzed as smoking related (n = 1251) and nonsmoking related (n = 1092). ^b^Other GI includes pancreas, small intestine, stomach, esophagus, other digestive organ cancers. Other genitourinary includes uterus, ovary, female breast, other female genital organs, other male genital organs, kidney, and bladder. Other hematologic includes acute and chronic leukemia, multiple myeloma, and other hematologic system cancers. Other skin includes basal, carcinoma not otherwise specified, skin trunk, and hemangiosarcoma. Incidence of each cancer less than 2% of the totals includes cervix, soft tissue, brain and other nervous system, thyroid, endocrine system, and eye and orbit.

The analytic sample of 3245 participants was drawn from a population of 5061 smokers referred during this time frame. As shown in Figure 1, 1816 individuals were excluded from this analysis as follows: (1) consultation initiated but no program treatment delivered owing to already being in treatment with another clinician or having incomplete consultation data, (2) no medical consultation provided, (3) used tobacco products other than cigarettes, (4) smoked less than 1 cigarette per day, (5) had a disease other than cancer, and (6) died before the 9-month follow-up assessment.

As shown in Figure 1, the analytical sample consisted of 2652 patients with a history of cancer that included both those with current cancer (n = 2343) and a history of cancer (n = 309), and 593 patients forming a no cancer history group comprising The University of Texas MD Anderson Cancer Center employees (n = 312) and cancer-screening patients (n = 281). Within the cancer history group, those with current cancer were divided into 9 cancer-site groupings, which was further classified as smoking-related or non–smoking-related cancers.

### Procedures

The TTP plan consisted of an initial in-person consultation (60-90 minutes), plus 6 to 8 subsequent follow-up treatment sessions (30-45 minutes) conducted over an 8- to 12-week period, 95% of which were conducted by telephone. Treatment involved behavioral counseling for smoking cessation and other psychological or psychiatric intervention, as needed, for related mental health issues. Counseling was based on principles of motivational interviewing^22^ and social cognitive behavioral problem solving.^23^ Patients typically received 10 to 12 weeks of pharmacotherapy including nicotine replacement (patch or lozenge), bupropion, and varenicline, either alone or in various combinations. Each treatment plan was personalized in terms of counseling session number, duration, content, and choice of pharmacotherapy, which followed a previously defined protocol^24^ consistent with National Comprehensive Cancer Network guidelines.^25^ The eMethods in the Supplement provides further details.

### Measures

At the initial consultation, psychiatric disorders were assessed using the Patient Health Questionnaire.^26^ Nicotine dependence was measured using the Fagerström Test for Cigarette Dependence (formerly the FTND) (score range, 0-10, with higher values indicating greater dependence).^27^ Cancer status (current cancer, previous cancer, and no cancer history) and cancer site were based on the MD Anderson Cancer Center Tumor Registry database and confirmed by review of patient medical records. Smoking relatedness of the cancer was based on the latest epidemiologic evidence.^1,28,29^ Race/ethnicity was self-identified.

Smoking status was assessed by support staff (noncounselors) using sequential timeline follow-back interviews^30^ at each contact and at 3-, 6-, and 9-month postconsultation follow-ups. The primary outcome for this study was timeline follow-back interview–determined, 7-day point-prevalence abstinence at 9 months, defined as self-report of no smoking (not even a puff) during the previous 7 days.

Given the clinical nature of our program, the varying health status of the patients, the time course of their cancer therapy, and the fact that only 5% of the sessions were conducted in person, requiring patients to return or otherwise provide biochemical assessment of abstinence was not feasible. However, we obtained expired carbon monoxide levels at all in-person visits.

### Statistical Analysis

Our overall analytical approach involved 2 major comparisons. First, to determine the outcome of ever having cancer associated with smoking abstinence, we compared all patients with a cancer history with those having no history of cancer (Figure 1). Second, to isolate the association of having a current cancer with abstinence, each site was compared with the group without a history of cancer.

We conducted bivariate comparisons of demographics and baseline variables for the major cancer groupings (cancer history, no cancer history, cancer site) using χ^2^ analysis for categorical comparisons and *t* tests for continuous comparisons. *P* values are 2-sided, with a significance level of <.05.

We used a modified Poisson generalized linear regression model^31,32^ (Stata, version 14; StataCorp) to evaluate differences in abstinence for the comparisons of cancer history vs no cancer history and no cancer history vs each current cancer site. Separate models were evaluated for each comparison at all time points (3, 6, and 9 months after consultation) that were both unadjusted and adjusted for the demographic and baseline covariates. Bonferroni corrections for multiple comparisons were applied by dividing 0.05 by the number of comparisons × number of time points within each model tested.

Additional secondary comparisons (eMethods in the Supplement) were carried out to assess the outcome associated with abstinence of having a smoking-related or smoking-unrelated cancer and of having a history of cancer. We also evaluated longitudinal models over 3 time points of abstinence for both major and secondary comparisons. Results of secondary comparisons for smoking-related and nonsmoking-related cancers and the longitudinal models for both the major and secondary comparisons are described in the eResults in the Supplement.

Regression analyses were carried out using 3 approaches to model missing smoking information. The analyses included multiple imputation, using existing covariates to estimate the missing abstinence data; intention-to-treat (ITT), imputing missing data as smoking; and respondent only, calculating abstinence rates on available assessments without imputation.

We present abstinence results using multiple imputation herein. Although it has been common practice to impute nonrespondents as smokers (ITT) in smoking cessation studies, this method may fail to account for the uncertainty of that imputation and gives a false sense of precision while ignoring other information that could contribute to the imputation.^33,34^ A multiple imputation approach attempts to account for this uncertainty. Results using both the ITT and respondent only approaches are reported in eTables 9-23 in the Supplement.

## Results

### Baseline Characteristics

Of the 3245 smokers, 1588 (48.9%) were men, 322 (9.9%) were of black race/ethnicity, 172 (5.3%) were of Hispanic race/ethnicity, and 2498 (76.0%) were of white race/ethnicity. Mean (SD) age was 54 (11.4) years; Fagerström Test for Cigarette Dependence score, 4.41 (2.2); number of cigarettes smoked per day, 17.1 (10.7); years smoked, 33 (13.2); and 1393 patients (42.9%) had at least 1 psychiatric comorbidity. Demographics for the sample of 3245 smokers are presented in Table 1 for cancer history (n = 2652) and no cancer history (n = 593), and breast (n = 266), colorectal and other gastrointestinal (includes pancreas, small intestine, stomach, esophagus, other digestive organ cancers) (n = 221), head and neck (n = 417), lung (n = 338), lymphoma and other hematologic (includes acute and chronic leukemia, multiple myeloma, and other hematologic system cancers) (n = 292), melanoma and other skin (includes basal, carcinoma not otherwise specified, skin trunk, and hemangiosarcoma) (n = 143), prostate (n = 110), other genitourinary (includes uterus, ovary, female breast, other female genital organs, other male genital organs, kidney, and bladder) (n = 275), and all other cancers (incidence of each cancer less than 2% of the totals; include cervix, soft tissue, brain and other nervous system, thyroid, endocrine system, and eye and orbit) (n = 281). In comparison with the no cancer history group, patients with a history of cancer were more likely to be older (mean [SD] age, 48.3 [12.3] vs 55.3 [10.8] years), male (237 [40.0%] vs 1351 [50.9%]), and of white race (321 [54.1%] vs 2177 [82.1%]); to have smoked more (median [interquartile range], 15 [10-20] vs 18 [10-20] cigarettes per day]) and longer (mean [SD], 27.0 [13.4] vs 34.5 [12.8] years); be more cigarette dependent based on the Fagerström Test for Cigarette Dependence score (mean [SD], 4.1 [2.2] vs 4.5 [2.2]; and have a higher incidence of current depression based on the Patient Health Questionnaire (142 of 571 [24.9%] vs 717 of 2433 [29.5%]). Compared with the group without cancer, a higher incidence of depression was noted for patients with breast cancer (34.7% vs 24.9%; *P* = .004), anxiety for patients with head and neck cancer (31.3% vs 24.2%; *P* = .03), and alcohol-related problems for patients with lung cancer (4.2% vs 10.5%; *P* = .001).

**Table 1.** Baseline Characteristics of 3245 Patients^a^

Characteristic	No Cancer History (n = 593)^a^	Cancer History (n = 2652)	Breast (n = 266)	Colorectal and Other GI (n = 221)^b^	Head Neck (n = 417)	Lung (n = 338)	Lymphoma and Other Hematologic (n = 292)^b^	Melanoma and Other Skin (n = 143)^b^	Prostate (n = 110)	Other Genitourinary (n = 275)^b^	Other Cancers (n = 281)^b^
Age, mean (SD), y	48.3 (12.3)	55.3 (10.8)	52.8(9.44)	56.9 (9.7)	55.6(10.1)	61.1 (9.2)	51.8 (11.8)	54.5 (11.5)	60.6 (7.15)	54.0 (11.0)	52.6 (11.3)
*P* value^c^		<.001	<.001	<.001	<.001	<.001	<.001	<.001	<.001	<.001	<.001
Men, No. (%)	237 (40.0)	1351 (50.9)	NA	144 (65.2)	287 (68.8)	169 (50.0)	178 (61.0)	81 (56.6)	110 (100)	129 (46.9)	130 (46.3)
*P* value^c^		<.001		<.001	<.001	.003	<.001	<.001	<.001	.05	.08
Race/ethnicity, No. (%)											
Black	71 (12.0)	251 (9.5)	41 (15.4)	17 (7.7)	28 (6.7)	36 (10.7)	28 (9.6)	2 (1.4)	11 (10.0)	20 (7.3)	25 (8.9)
Hispanic	29 (4.9)	143 (5.4)	18 (6.8)	12 (5.4)	19 (4.6)	9 (2.7)	24 (8.2)	4 (2.8)	6 (5.5)	18 (6.5)	17 (6.0)
Other	172 (29.0)	81 (3.1)	7 (2.6)	14 (6.3)	9 (2.2)	9 (2.7)	14 (4.8)	1 (0.7)	3 (2.7)	5 (1.8)	11 (3.9)
White	321 (54.1)	2177 (82.1)	200 (75.2)	178 (80.5)	361 (86.6)	284 (84.0)	226 (77.4)	136 (95.1)	90 (81.8)	232 (84.4)	228 (81.1)
*P* value^c^		<.001	<.001	<.001	<.001	<.001	<.001	<.001	<.001	<.001	<.001
Psychiatric Comorbidities on PHQ, No. (%)											
No	318 (56.1)	1239 (51.0)	111 (44.9)	104 (53.06)	175 (46.1)	169 (54.9)	129 (53.8)	75 (56.4)	62 (62.6)	126 (54.3)	117 (47.2)
Yes	249 (43.9)	1144 (48.0)	136 (55.1)	92 (46.9)	205 (54.0)	139 (45.1)	111 (46.3)	58 (43.6)	37 (37.4)	106 (45.7)	131 (52.8)
*P* value^c^		.08	.003	.46	.002	.73	.54	.95	.22	.65	.02
Anxiety, No. (%)											
No	433 (75.8)	1796 (74.0)	177 (69.7)	149 (74.1)	266 (68.7)	229 (73.6)	181 (73.6)	105 (78.4)	84 (83.2)	180 (74.1)	173 (68.4)
Yes	138 (24.2)	635 (26.1)	77 (30.3)	52 (25.9)	121 (31.3)	82 (26.4)	65 (26.4)	29 (21.6)	17 (16.8)	63 (25.9)	80 (31.6)
*P* value^c^		.34	.06	.93	.03	.37	.45	.79	.22	.95	.06
Alcohol-related											
No	511 (89.5)	2201 (90.5)	228 (89.8)	182 (90.6)	344 (88.9)	298 (95.8)	225 (91.5)	117 (87.3)	84 (83.2)	225 (92.6)	224 (88.5)
Yes	60 (10.5)	230 (9.5)	26 (10.2)	19 (9.5)	43 (11.1)	13 (4.2)	21 (8.5)	17 (12.7)	17 (16.8)	18 (7.4)	29 (11.5)
*P* value^c^		.45	.91	.67	.77	.001	.39	.47	.07	.17	.68
Depression											
No	429 (75.1)	1716 (70.5)	166 (65.4)	138 (68.7)	265 (68.5)	220 (70.5)	182 (73.)	102 (76.1)	83 (82.2)	175 (72.0)	167 (66.0)
Yes	142 (24.9)	717 (29.5)	88 (34.7)	63 (31.3)	122 (31.5)	92 (29.5)	65 (26.3)	32 (23.9)	18 (17.8)	68 (28.0)	86 (34.0)
*P* value^c^		.03	.004	.07	.02	.14	.66	.81	.12	.35	.007
Smoking cessation medication, No. (%)											
No	23 (3.9)	156 (5.9)	15 (5.6)	14 (6.3)	46 (11.0)	9 (2.7)	16 (5.5)	4 (2.8)	5 (4.5)	13 (4.7)	14 (5.0)
Yes	570 (96.1)	2496 (94.1)	25 1 (94.4)	207 (93.67)	371 (88.97)	329 (97.3)	276 (94.5)	139 (97.2)	105 (95.5)	262 (95.3)	267 (95.0)
*P* value^c^		.05	.25	.13	<.001	.33	.27	.54	.74	.56	.45
FTCD score, mean (SD)^d^	4.1 (2.2)	4.5 (2.2)	4.14 (2.18)	4.47 (2.04)	4.70 (2.25)	4.60 (2.05)	4.21 (2.19)	4.96 (2.20)	4.29 (2.36)	4.96 (2.29)	4.56 (2.20)
*P* value		<.001	.68	.03	<.001	<.001	.41	<.001	.37	<.001	.004
CPD, median (IQR)	15 (10-20)	18 (10-20)	15 (10-20)	16 (10-20)	20 (10-20)	18 (9-20)	15 (10-20)	20 (11-20)	20 (10-30)	20 (10-25)	20 (10-20)
*P* value^c^^,^^e^		.001	>.99	.47	<.001	.01	>.99	.002	.006	<.001	<.001
Years smoked, mean (SD)	27.0 (13.4)	34.5 (12.8)	30.6 (10.9)	36.4 (11.8)	35.3 (12.2)	41.81 (10.8)	31.29 (13.4)	33.5 (12.8)	37. (11.8)	33.9 (13.3)	32.8 (12.6)
*P* value^c^		<.001	<.001	<.001	<.001	<.001	<.001	<.001	<.001	<.001	<.001

Abbreviations: CPD, cigarettes per day; FTCD, Fagerström Test for Cigarette Dependence; GI, gastrointestinal; IQR, interquartile range; PHQ, Patient Health Questionnaire.

^a^ Denominators vary in some areas where data were not available for all patients.

^b^ The other listing for each type of cancer is presented in the first paragraph of the Results section.

^c^ All *P* values are based on 2-tailed *t* tests for continuous and χ^2^ test for categorical comparisons of a cancer group (cancer history, current sites) vs the no cancer history group.

^d^ Possible range, 0 to 10; higher scores indicate greater dependence.

^e^ *P* values were estimated with quantile regression.

Response rates for abstinence data were 92.4% at 3 months, 86.7% at 6 months, and 81.9% at 9 months for the cancer history group, and were 91.1% at 3 months, 83.6% at 6 months, and 76.6% at 9 months for the no cancer history group. Baseline characteristics for the following analytical groups are presented in eTable1 in the Supplement: patients with and without a cancer history, those without a history of cancer, and those with smoking-related and smoking-unrelated cancers.

### Overall Abstinence

**Figure 2.**
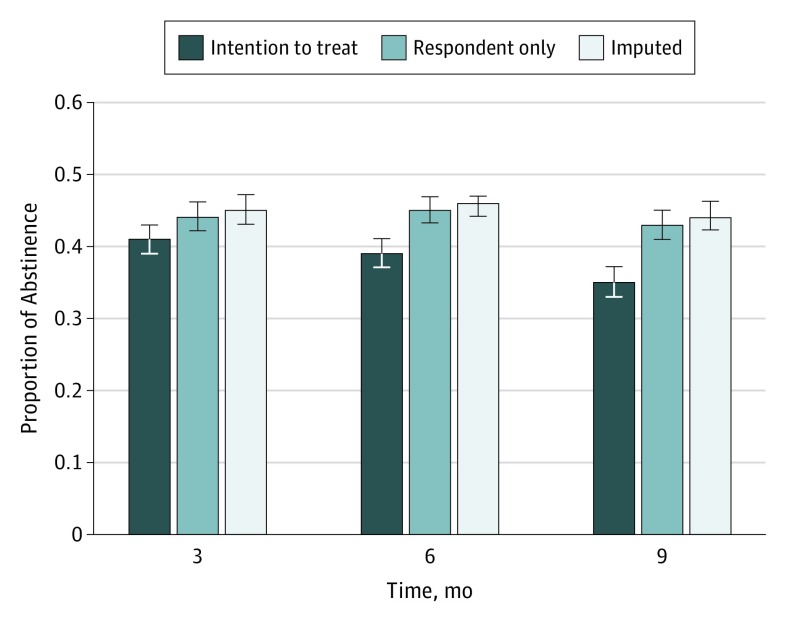
Proportion of Abstinence Abstinence for all 3 time points by intention-to-treat, respondent-only, and multiply imputed samples. Error bars indicate 95% CIs.

Overall self-reported abstinence rates for the sample were 45.1% at 3 months, 45.8% at 6 months, and 43.7% at 9 months for the multiply imputed data (averaged over 10 imputed data sets) (eTable 2 in the Supplement); 41.1% at 3 months, 39.5% at 6 months, and 35.6% at 9 months for ITT; and 44.5% at 3 months, 45.6% at 6 months, and 43.7% at 9 months for respondent only (Figure 2). Overall abstinence rates by cancer site and cancer history are reported in Table 2. As noted above, we obtained expired carbon monoxide levels at all in-person visits. There was a total of 8877 of such biochemical assessments of abstinence. Congruence between self-reported, 7-day point prevalence abstinence and expired carbon monoxide was 93% for less than 8 ppm and 87% for carbon monoxide level of less than 6 ppm.

**Table 2.** Abstinence by Cancer Category (N = 3245)^a^

Characteristic	No Cancer History (n = 593)	Cancer History (n = 2652)	Breast (n = 266)	Colorectal and Other GI (n = 221)^b^	Head and Neck (n = 417)	Lung (n = 338)	Lymphoma and Other Hematologic (n = 292)^b^	Melanoma and Other Skin (n = 143)^b^	Prostate (n = 110)	Other Genitourinary (n = 275)^b^	Other Cancers (n = 281)^b^
**Multiply Imputed, %**											
3 mo	43.8	45.5	44.1	41.8	52.2	50.3	44.6	42.5	43.1	43.3	43.2
6 mo	44.3	46	48.3	44.5	53.3	49.2	40.1	43.4	40.1	41.7	45.7
9 mo	41.5	44.6	52.1	41.2	53.6	48.1	39.5	42.1	37.6	39.5	43.4
**Intention-to-Treat**											
3 mo	39.5	41.4	39.1	37.6	46.3	46.4	41.8	39.9	42.7	36	37.8
6 mo	36.8	39.9	40.2	38.9	44.8	43.5	36.0	35.7	39.1	33.4	39.1
9 mo	31.5	36.5	39.1	34.4	42.2	39.6	33.6	33.6	31.5	30.2	35.9
**Respondent Only, Total No. (%)**											
3 mo	540 (43.3)	2452 (44.8)	252 (41.3)	203 (40.9)	375 (51.5)	319 (49.2)	274 (44.5)	136 (41.9)	99 (47.5)	249 (39.8)	258 (41.1)
6 mo	496 (43.9)	2306 (45.8)	237 (45.1)	189 (45.5)	346 (54.0)	304 (48.4)	254 (41.3)	127 (40.2)	94 (45.7)	236 (39.0)	245 (44.9)
9 mo	454 (41.2)	2189 (44.3)	220 (47.3)	183 (41.5)	322 (54.7)	289 (46.4)	238 (41.2)	122 (39.3)	88 (38.6)	225 (36.9)	232 (43.5)

Abbreviation: GI, gastrointestinal.

^a^ Denominators vary in some areas where data were not available for all patients.

^b^ The other listing for each type of cancer is presented in the first paragraph of the Results section.

### Comparisons Across Cancer Groupings

**Figure 3.**
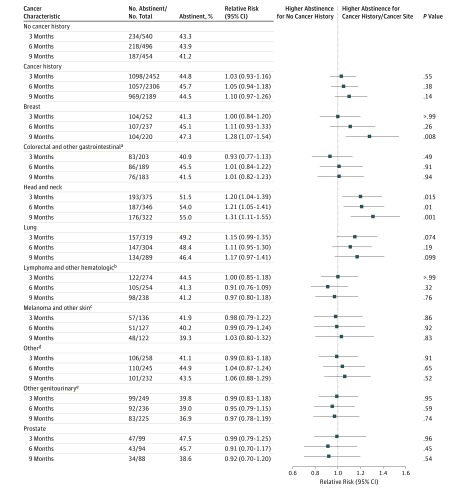
Adjusted Models in the Multiply Imputed Sample No history of cancer served as the reference category. Comparisons: the overall effect of ever having cancer and the association of specific cancer sites with abstinence at the 3-, 6-, and 9-month follow-ups. Reference group for both comparisons is the no cancer history group. Nominal *P* values are shown; multiple correction *P* values over 3 time points are .002 for cancer site vs no cancer history and .02 for cancer history vs no cancer history. Cancer history includes patients with current cancer; no cancer history includes employees and screening patients. ^a^Other gastrointestinal includes pancreas, small intestine, stomach, esophagus, and other digestive organs. ^b^Other hematologic includes acute and chronic leukemia, multiple myeloma, and other hematologic system cancers. ^c^Other skin includes basal carcinoma not otherwise specified, skin trunk, and hemangiosarcoma. ^d^Other includes cervix, soft tissue, brain and other nervous system, thyroid, endocrine system, and eye and orbit. ^e^Other genitourinary includes uterus, ovary, female breast, other female genital organs, other male genital organs, kidney, and bladder.

As presented in Figure 3 and eTable 3 in the Supplement, no significant differences in abstinence for the multiply imputed sample were found when comparing no cancer history vs cancer history at the 3-month (relative risk [RR], 1.03; 95%CI, 0.93-1.16; *P* = .55), 6-month (RR, 1.05; 95% CI, 0.94-1.18; *P* = .38), and 9-month (RR, 1.10; 95% CI, 0.97-1.26; *P* = .14) follow-ups, as well as in the longitudinal models (RR, 1.06, 95% CI, 0.95-1.18; *P* = .27) (eFigure 1, eTable 4 in the Supplement). In addition, no significant differences were noted in the comparisons of no cancer history vs those with and without smoking-related cancer (RR, 1.02; 95% CI, 0.90-1.14; *P* = .8) or patients with a history of cancer (RR, 1.04; 95% CI, 0.89-1.20; *P* = .64) (eFigure 2 in the Supplement). The ITT and respondent-only results were largely consistent with the multiply imputed results and showed no significant differences for these same comparisons (eTables 9-12 in the Supplement) that survived correction for multiple comparisons (*P* < .02), with the exception of higher abstinence at 9 months for the cancer history vs no history group (eTable 9 in the Supplement) comparison in the ITT-adjusted model (RR, 1.20; 95% CI, 1.04-1.39; *P* = .01).

Among individual cancer sites, only the head and neck cancer group at 9 months (RR, 1.31; 95% CI, 1.11-1.56; *P* = .001) (Figure 3) and in the longitudinal model (RR, 1.24; 95% CI, 1.08-1.42; *P* = .002) (eFigure 1 in the Supplement), abstained more often than the no cancer history group in the multiply imputed sample, when correcting for multiple comparisons. None of the other secondary comparisons across cancer sites were significant (eTables 17-19 in the Supplement). In addition, none of the supplementary comparisons between the no cancer history group and those with and without smoking-related cancers or a history of cancer was significant when correcting for multiple comparisons (*P* < .006) using the multiply imputed (eTables 5-8 in the Supplement) ITT or respondent-only (eTables 13-16; eTables 20-23 in the Supplement) samples.

## Discussion

This study examined whether a comprehensive TTP that uses personalized intensive counseling and proactive pharmacologic management by cancer history or cancer site was associated with smoking cessation among patients with cancer and whether cessation can be sustained. We used both time-specific (3-, 6-, and 9-month follow-ups) and longitudinal covariate-adjusted and unadjusted models with multiple imputation, ITT, and respondent-only approaches to evaluate missing abstinence data. Overall results across all models were consistent and suggest that, in comparison with smokers with no cancer history, abstinence rates within our TTP program did not appear to differ appreciably whether smokers had current cancer, a history of cancer, are a cancer survivor, or had smoking-related or nonsmoking-related cancers. Abstinence rates by individual cancer site did not differ in comparison with those in the no cancer history group with the exception of patients with head and neck cancer in some analyses.

Although we observed some decline in abstinence for those with a cancer history from 3 to 9 months in the ITT sample (from 41.1% to 35.6%) the respondent-only and the multiply imputed models suggest a consistent level of program abstinence over time of approximately 45%. The similarity between multiply imputed and respondent-only results also suggests that these methods may be more appropriate missing-data adjustments for this population than the ITT approach. Factors such as having already quit or worsening health, for example, may account for missingness as opposed to reluctance to participate in follow-ups owing to smoking, which is the presumption in the ITT approach.

It is difficult to compare absolute abstinence rates across studies without a placebo or control group that can be used to judge effect sizes across settings and over time. Such control groups would be rare in clinical cohort studies such as ours. That notwithstanding, the size of our sample and the consistency of our results suggest that, with comprehensive treatment, nearly half of the patients with cancer can be expected to quit and maintain abstinence following initial exposure to treatment. This finding contrasts with the less-robust long-term (6-month) finding (22%) observed in a meta-analysis of cessation studies in patients with cancer receiving less-intensive interventions.^18^

Our data suggest that better results could be expected with full integration of comprehensive tobacco treatment into the oncologic setting. However, adoption of this approach has often been limited by barriers related to information technology, time and training of personnel, costs, and poor reimbursement. We acknowledge that the MD Anderson Cancer Center program is unique in that it is funded primarily through Texas Tobacco Settlement Funds awarded to the state as part of the Tobacco Master Settlement. However, this progressive undertaking could serve as a model and impetus for other states to examine how tobacco treatment is funded within state-supported health care systems. The MD Anderson Cancer Center TTP uses a single in-person contact to conduct an initial assessment for treatment planning, including a medical evaluation for pharmacotherapy selection. Moreover, 95% of the counseling sessions are conducted by telephone, and the program now uses video technology, so that even the face-to-face, in-person session can be conducted remotely. Minimizing face-to-face encounters, while maintaining quality, could put such a program within reach of other cancer centers where size and resources allow.

Presently, many cancer centers and other health care institutions rely heavily on telephone quitlines for smoking cessation, which seems to be well reasoned in low-resource settings. Although quitlines are clearly an important component of a national tobacco control strategy, given their low cost and broad reach, a more comprehensive approach may be required to maximize abstinence for patients with a life-threatening illness, such as cancer, particularly those with psychiatric comorbidities and other significant psychosocial stressors. Although one recent study did not focus on patients with cancer, the findings showed that the average quitline ITT abstinence rates for those with and without mental health disorders (excluding bipolar and schizophrenia) were 9% and 14%, respectively, at a 7-month follow-up.^35^

Considering the expense of treating complex medical disorders, especially cancer, where costs of $157.7 billion are projected by 2020,^36^ the downstream value of an effective smoking cessation program cannot be understated. Cost-effectiveness of smoking cessation has been well documented in the general population^37^ and among patients with cancer.^38,39,40^ Although more-intensive interventions are associated with greater success, the absolute cost is higher than that of minimal interventions.^41^ Nevertheless, investment in a comprehensive program within cancer centers may be justified by the potential savings in treatment costs (reduced mortality, second primary cancers, progression, recurrence), and by the improvements in treatment response^1^ and quality of life. Patients with cancer should be given the best opportunity to achieve cessation, just as we select the most effective medical treatments to increase their likelihood of survival. The costs for achieving smoking cessation are less consequential than the costs of cancer treatment failure, complications, and toxic effects due to a patient’s inability to quit smoking. For example, the average annual program costs to treat new and returning patients is $800 to $1000, with a cost per quit of $1900 to $2500, which, in the context of cancer care, seems well justified.^40^

### Limitations

This study has limitations. This study was not a randomized clinical trial comparing our comprehensive approach with minimal care or other less-intensive interventions. Such studies appear to be needed to determine the long-term cost-effectiveness of comprehensive treatment for patients with serious medical illnesses, such as cancer. Nevertheless, it seems that both ethical and societal benefit arguments can be made for giving patients the best opportunity for survival by maximizing their chances of achieving tobacco cessation.

We also did not examine the contributions of individual treatment components, such as medication, or outcome differences among patients with psychiatric comorbidities, although we controlled for these factors in our statistical analyses. This article focused on overall program outcome. Examination of these other factors will be the subject of future reports.

In addition, our abstinence results were not uniformly biochemically verified, although concordance between in-person self-report and expired carbon monoxide levels was as high as 93% and above that reported in other studies of patients with cancer.^42^ Biochemical verification in large clinical samples where most interaction is remote does not seem to be generally feasible, nor does it appear to be necessary if we accept the same standard as quitlines, which do not verify abstinence.^43^

## Conclusions

This study evaluated the smoking abstinence rates associated with a comprehensive TTP in a large sample (N = 3245) and found mean 7-day smoking abstinence rates of 45.1% at the 3-month follow-up, 45.8% at the 6-month follow-up, and 43.7% at the 9-month follow-up. Rates did not differ between patients with cancer and those without cancer. Our study results suggest that providing comprehensive tobacco treatment in the oncologic setting may result in sustained high abstinence rates for all patients with cancer and survivors and recommend that this intervention be included as standard of care to ensure the best possible cancer treatment outcomes.

## Notes

Supplement. **eMethods.** Detailed Methodology **eResults.** Demographics and Models **eTable 1.** Baseline Characteristics of Patients **eTable 2.** Overall Abstinence **eTable 3.** Unadjusted and Adjusted Multiply Imputed Results for Cancer History (CaHx+) vs. No -Cancer History (CaHx-) Categories for Each Time Point **eTable 4.** Unadjusted and Adjusted Multiply Imputed Results for Cancer History (CaHx+) vs. No -Cancer History (CaHx-) Categories in Longitudinal Analyses **eTable 5.** Unadjusted and Adjusted Multiply-Imputed Results for Smoking-Related (SMCa+) and Non-Smoking Related Cancer (SMCa-) vs. No Cancer History (Cahx-) and vs. Each Other for Each Time Point **eTable 6.** Unadjusted and Adjusted Multiply-Imputed Results for Smoking-Related (SMCa+) and Non-Smoking Related Cancer (SMCa-) vs. No Cancer History (Cahx-) and vs. Each Other for Longitudinal Analyses **eTable 7.** Unadjusted and Adjusted Multiply Imputed Results for Cancer Survivor (S) vs No Cancer History (Cahx-) and Current Cancer-All Sites (Cca) For Each Time Point **eTable 8.** Unadjusted and Adjusted Multiply Imputed Results for Cancer Survivor (S) vs No Cancer History (Cahx-) and Current Cancer-All Sites (Cca) for Longitudinal Analyses **eTable 9.** Unadjusted and Adjusted ITT Results for Cancer History (CaHx+) vs. No -Cancer History (CaHx-) Categories for Each Time Point **eTable 10.** Unadjusted and Adjusted Respondent-Only Results for Cancer History (CaHx+) vs. No -Cancer History (CaHx-) Categories for Each Time Point **eTable 11.** Unadjusted and Adjusted ITT Results for Cancer History (CaHx+) vs. No -Cancer History (CaHx-) Categories for the Longitudinal Analysis **eTable 12.** Unadjusted and Adjusted Respondent-Only Results for Cancer History (CaHx+) vs. No -Cancer History (CaHx-) Categories for the Longitudinal Analysis **eTable 13.** Unadjusted and Adjusted ITT Results for Smoking-Related (SMCa+) and Non-Smoking Related (SMCa-) Cancer vs. No Cancer History (Cahx-) and vs. Each Other for Each Time Point **eTable 14.** Unadjusted and Adjusted Respondent-Only Results for Smoking-Related (SMCa+) and Non-Smoking Related (SMCa-) Cancer vs. No Cancer History (Cahx-) and vs. Each Other for Each Time Point **eTable 15.** Unadjusted and Adjusted ITT Results for Smoking-Related (SMCa+) and Non-Smoking Related (SMCa-) Cancer vs. No Cancer History (Cahx-) and vs. Each Other for Each Time Point **eTable 16.** Unadjusted and Adjusted Respondent-Only Results for Smoking-Related vs Non-Smoking Related Categories for Longitudinal Analyses **eTable 17.** Unadjusted and Adjusted Multiply-Imputed Results for Cancer Site Categories vs. No Cancer History (CaHx-) **eTable 18. **Unadjusted and Adjusted ITT Results for Cancer Site Categories vs. No Cancer History (CaHx) **eTable 19.** Unadjusted and Adjusted Respondent-Only Results for Cancer Site Categories vs. No Cancer History (Cahx-) for Each Time Point **eTable 20.** Unadjusted and Adjusted ITT Results for Cancer Survivor (S) vs. No Cancer (CaHx-) and Current Cancer (CCa) for Each Time Point **eTable 21.** Unadjusted and Adjusted Respondent-Only Results for Cancer Survivor (S) vs. No Cancer (CaHx-) and Current Cancer (CCa) for Each Time Point **eTable 22.** Unadjusted and Adjusted ITT Results for Cancer Survivor (S) vs. No Cancer (CaHx-) and Current Cancer (CCa) **eTable 23.** Unadjusted and Adjusted Respondent-Only Results for Cancer Survivor (S) vs. No Cancer (CaHx-) and Current Cancer (CCa) **eFigure 1.** Longitudinal Adjusted Models of the Impact of Ever Having Cancer (CAHx+); Having a Smoking Related (SMCA+) or Unrelated (SMCA-) Cancer; Being a Cancer Survivor (S) or of Individual Cancer Site on Abstinence Over Time Compared to Never Having Cancer (Cahx-) **eFigure 2.** Adjusted Models of the Impact of Smoking Related Cancer and Survivor Groups on Abstinence Compared to No Cancer Group (CaHx-) by Month and Longitudinally for the Multiply Imputed Sample **eReferences**
