# Supplementary material for: Association of a Comprehensive Smoking Cessation Program With Smoking Abstinence Among Patients With Cancer
Source: JAMA Netw Open. 2019 Sep 27;2(9):e1912251. doi: 10.1001/jamanetworkopen.2019.12251 (PMC6777393; doi:10.1001/jamanetworkopen.2019.12251)
Supplement: Supplement. — eMethods. Detailed Methodology eResults. Demographics and Models eTable 1. Baseline Characteristics of Patients eTable 2. Overall Abstinence eTable 3. Unadjusted and Adjusted Multiply Imputed Results for Cancer History (CaHx+) vs. No -Cancer History (CaHx-) Categories for Each Time Point eTable 4. Unadjusted and Adjusted Multiply Imputed Results for Cancer History (CaHx+) vs. No -Cancer History (CaHx-) Categories in Longitudinal Analyses eTable 5. Unadjusted and Adjusted Multiply-Imputed Results for Smoking-Related (SMCa+) and Non-Smoking Related Cancer (SMCa-) vs. No Cancer History (Cahx-) and vs. Each Other for Each Time Point eTable 6. Unadjusted and Adjusted Multiply-Imputed Results for Smoking-Related (SMCa+) and Non-Smoking Related Cancer (SMCa-) vs. No Cancer History (Cahx-) and vs. Each Other for Longitudinal Analyses eTable 7. Unadjusted and Adjusted Multiply Imputed Results for Cancer Survivor (S) vs No Cancer History (Cahx-) and Current Cancer-All Sites (Cca) For Each Time Point eTable 8. Unadjusted and Adjusted Multiply Imputed Results for Cancer Survivor (S) vs No Cancer History (Cahx-) and Current Cancer-All Sites (Cca) for Longitudinal Analyses eTable 9. Unadjusted and Adjusted ITT Results for Cancer History (CaHx+) vs. No -Cancer History (CaHx-) Categories for Each Time Point eTable 10. Unadjusted and Adjusted Respondent-Only Results for Cancer History (CaHx+) vs. No -Cancer History (CaHx-) Categories for Each Time Point eTable 11. Unadjusted and Adjusted ITT Results for Cancer History (CaHx+) vs. No -Cancer History (CaHx-) Categories for the Longitudinal Analysis eTable 12. Unadjusted and Adjusted Respondent-Only Results for Cancer History (CaHx+) vs. No -Cancer History (CaHx-) Categories for the Longitudinal Analysis eTable 13. Unadjusted and Adjusted ITT Results for Smoking-Related (SMCa+) and Non-Smoking Related (SMCa-) Cancer vs. No Cancer History (Cahx-) and vs. Each Other for Each Time Point eTable 14. Unadjusted and Adjusted Respondent-Only R [file jamanetwopen-2-e1912251-s001.pdf]

Supplementary Online Content

Cinciripini PM, Karam-Hage M, Kypriotakis G, et al. Association of a comprehensive smoking cessation program with smoking abstinence among patients with cancer. *JAMA Netw Open*. 2019;2(9):e1912251. doi:10.1001/jamanetworkopen.2019.12251

eMethods. Detailed Methodology

eResults. Demographics and Models

eTable 1. Baseline Characteristics of Patients

eTable 2. Overall Abstinence

eTable 3. Unadjusted and Adjusted Multiply Imputed Results for Cancer History (CaHx+) vs. No -Cancer History (CaHx-) Categories for Each Time Point

eTable 4. Unadjusted and Adjusted Multiply Imputed Results for Cancer History (CaHx+) vs. No -Cancer History (CaHx-) Categories in Longitudinal Analyses

eTable 5. Unadjusted and Adjusted Multiply-Imputed Results for Smoking-Related (SMCa+) and Non-Smoking Related Cancer (SMCa-) vs. No Cancer History (Cahx-) and vs. Each Other for Each Time Point

eTable 6. Unadjusted and Adjusted Multiply-Imputed Results for Smoking-Related (SMCa+) and Non-Smoking Related Cancer (SMCa-) vs. No Cancer History (Cahx-) and vs. Each Other for Longitudinal Analyses

eTable 7. Unadjusted and Adjusted Multiply Imputed Results for Cancer Survivor (S) vs No Cancer History (Cahx-) and Current Cancer-All Sites (Cca) For Each Time Point

eTable 8. Unadjusted and Adjusted Multiply Imputed Results for Cancer Survivor (S) vs No Cancer History (Cahx-) and Current Cancer-All Sites (Cca) for Longitudinal Analyses

eTable 9. Unadjusted and Adjusted ITT Results for Cancer History (CaHx+) vs. No -Cancer History (CaHx-) Categories for Each Time Point

eTable 10. Unadjusted and Adjusted Respondent-Only Results for Cancer History (CaHx+) vs. No -Cancer History (CaHx-) Categories for Each Time Point

eTable 11. Unadjusted and Adjusted ITT Results for Cancer History (CaHx+) vs. No -Cancer History (CaHx-) Categories for the Longitudinal Analysis

eTable 12. Unadjusted and Adjusted Respondent-Only Results for Cancer History (CaHx+) vs. No -Cancer History (CaHx-) Categories for the Longitudinal Analysis

eTable 13. Unadjusted and Adjusted ITT Results for Smoking-Related (SMCa+) and Non-Smoking Related (SMCa-) Cancer vs. No Cancer History (Cahx-) and vs. Each Other for Each Time Point

eTable 14. Unadjusted and Adjusted Respondent-Only Results for Smoking-Related (SMCa+) and Non-Smoking Related (SMCa-) Cancer vs. No Cancer History (Cahx-) and vs. Each Other for Each Time Point

eTable 15. Unadjusted and Adjusted ITT Results for Smoking-Related (SMCa+) and Non-Smoking Related (SMCa-) Cancer vs. No Cancer History (Cahx-) and vs. Each Other for Each Time Point

eTable 16. Unadjusted and Adjusted Respondent-Only Results for Smoking-Related vs Non-Smoking Related Categories for Longitudinal Analyses

eTable 17. Unadjusted and Adjusted Multiply-Imputed Results for Cancer Site Categories vs. No Cancer History (CaHx-)

eTable 18. Unadjusted and Adjusted ITT Results for Cancer Site Categories vs. No Cancer History (CaHx)

eTable 19. Unadjusted and Adjusted Respondent-Only Results for Cancer Site Categories vs. No Cancer History (Cahx-) for Each Time Point

eTable 20. Unadjusted and Adjusted ITT Results for Cancer Survivor (S) vs. No Cancer (CaHx-) and Current Cancer (CCa) for Each Time Point

eTable 21. Unadjusted and Adjusted Respondent-Only Results for Cancer Survivor (S) vs. No Cancer (CaHx-) and Current Cancer (CCa) for Each Time Point

eTable 22. Unadjusted and Adjusted ITT Results for Cancer Survivor (S) vs. No Cancer (CaHx-) and Current Cancer (CCa)

eTable 23. Unadjusted and Adjusted Respondent-Only Results for Cancer Survivor (S) vs. No Cancer (CaHx-) and Current Cancer (CCa)

eFigure 1. Longitudinal Adjusted Models of the Impact of Ever Having Cancer (CAHx+); Having a Smoking Related (SMCA+) or Unrelated (SMCA-) Cancer; Being a Cancer Survivor (S) or of Individual Cancer Site on Abstinence Over Time Compared to Never Having Cancer (Cahx-)

eFigure 2. Adjusted Models of the Impact of Smoking Related Cancer and Survivor Groups on Abstinence Compared to No Cancer Group (CaHx-) by Month and Longitudinally for the Multiply Imputed Sample

eReferences.

This supplementary material has been provided by the authors to give readers additional information about their work.

© 2019 Cinciripini, PM, et al. *JAMA Network Open.*

eMethods. Detailed Methodology

Procedures Detail for the Tobacco Treatment Program

The initial consultation was conducted by a doctoral- or masters-level counselor to whom the participant was assigned for the duration of the program. This visit was 60 to 90 minutes in duration and included a detailed history on smoking, nicotine dependence, cancer treatment, psychosocial factors, alcohol use, psychiatric disorders (including depression and anxiety), as well as other family and environmental factors (e.g., financial stress, family support) that might be relevant to treatment. The program physician and/or medical staff (physician assistant) conducted a medical review to determine the appropriateness of specific smoking cessation medications. The majority of participants were offered 12 weeks of smoking cessation medication comprised of varenicline, bupropion, nicotine replacement (NRT; patch, gum, lozenge, inhaler, or spray), or a combination of these, taking into consideration past use and success on various pharmacotherapies, presence of contraindications for certain medications, and participant preference. The choice of medication following a clinical protocol have been published elsewhere[1].

Following the initial consultation, participants were scheduled for 6 to 8 follow-up counseling sessions over the next 8-12 weeks. The follow-up sessions were approximately 30 to 45 minutes in duration. Our protocol allowed participants to receive additional sessions if clinically warranted (e.g., participant had not quit yet but continued to be motivated to do so). Sessions were conducted over the phone and/or in person depending on the participant's location, treatment schedule, individual preferences, and level of progress.

The framework for smoking cessation counseling was based on principles of motivational interviewing[2] and social cognitive behavioral problem-solving–based strategies[3]. The specific components of the counseling sessions included those recommended by the Clinical Practice Guideline[4] for treating nicotine dependence (e.g., problem solving skills training and intra-treatment social support), as well as the use of motivational strategies for patients who were ambivalent about making changes in their smoking behavior. Having a quit-date was not a requirement for treatment entry. Counselors also addressed stressors associated with cancer treatment, family, finances, etc., as potential barriers to abstinence by providing supportive listening and assistance with problem solving (copy of treatment manual available upon request).

While mental health concerns were incorporated into the smoking cessation counseling provided by the TTP counselors, consultation by the program addiction psychiatrist was available for evaluation and treatment of severe psychiatric symptoms (e.g., depression, sleep disturbance, heavy alcohol use, and anxiety) that posed significant cognitive

© 2019 Cinciripini, PM, et al. *JAMA Network Open.*

and functional barriers to the participants' continued progress toward smoking cessation. This occurred in about 4% of the sample.

The initial consultations and medical evaluation was conducted in a face-to-face encounter at MD Anderson. However, to meet the needs of the patient, follow-up counseling sessions could be conducted either in-person or over the telephone. Approximately two-thirds of our patients lived outside the Houston metropolitan area, making travel to the center problematic. Where possible, an in-person follow-up visit was scheduled to coincide with return medical visits, but approximately 95% of the follow-up counseling sessions for the analytical sample were conducted by phone.

Medication and Counseling Sessions

Of the 3,245 patients included in this analysis, 167 (5.15%) elected not to receive medication. Among the 3,078 that did, the distribution of medication at week 12 was as follows: 278 (9%) bupropion; 1392 (43%) varenicline; 1192 (37%) single or combination NRT (i.e., patch plus lozenge); 127 (4%) bupropion and NRT; 56 (2%) received bupropion and varenicline; 10 (0.3%) received, NRT and varenicline; and finally 23 (0.6%) received some other treatment. The average number of follow-up sessions was 6.0 (SD=4.4).

Measures

Psychiatric disorders were assessed at the consultation visit using the Patient Health Questionnaire (PHQ)[5], a self-report questionnaire derived from the PRIME-MD (Primary Care Evaluation of Mental Disorders)[6] that assesses 8 of the most common DSM-IV Axis I disorders seen in primary care. The PHQ has diagnostic validity comparable to the original clinician-administered PRIME-MD. Diagnoses made with the PHQ have been found to be related to increases in health care utilization and functional impairment[7,8]. Only major depressive disorder, anxiety and panic disorders, and probable alcohol abuse modules were administered in this program. For simplicity, "anxiety disorders" is used here to refer to the category of anxiety and panic disorders and "alcohol abuse" is used to refer to the probable alcohol abuse category in DSM-IV[9].

Severity of nicotine dependence was measured at the consultation session with the Fagerström Test for Cigarette Dependence (FTCD), a 6-item questionnaire that assesses various components of smoking behavior[10].

Cancer status (current cancer patient, survivor, and no cancer history) and cancer site were based on information in the MD Anderson Tumor Registry database (TRDB) that was corroborated and updated by reviewing current information in the patient medical record. The TRDB codes all malignancies over the life of the patient, including benign neoplasms seen at MD Anderson, and non-neoplastic conditions that might affect the patient's cancer treatment. Information is abstracted from the chart (paper and/or electronic) at least 4 months but not longer than 6 months after

© 2019 Cinciripini, PM, et al. *JAMA Network Open.*

the patient's initial registration. The time lapse allows definitive diagnoses to have been made and the first course of treatment to have been given. The TRDB also does annual vital status follow-up on all eligible cancer patients. Quality control of abstracted information includes computerized edits for all applicable data items and verification by a second coder of cancer site, histology, and stage.

Smoking status was assessed at the initial TTP consultation and throughout the entire treatment and follow-up period by sequential Timeline Follow-Back interviews[11]. For this study, we used TLFB data to determine 7-day point-prevalence abstinence at 3 months, 6 months, and 9 months after initial consultation. These abstinence assessments were separate from the counseling sessions and conducted by support staff, not involved in counseling. Abstinence was defined as a self-report of no smoking (not even a puff) during the previous 7 days.

Since our study is not randomized we needed to adjust for important covariates in order to reduce potential confounding, and consequently bias, in our estimates. In observational studies to properly estimate the effect of a treatment on an outcome we need to adjust for covariates[12]. To reduce bias and adjust for pre-treatment characteristics in our comparisons groups we included covariates that capture demographic, psychosocial, pharmacotherapy, smoking related, and cancer specific characteristics, factors that have been shown[13–16] to affect abstinence rates. Note, however, that we replicated all analyses without adjustment for covariates to evaluate the sensitivity of the results when including or excluding the covariates from the analyses.

© 2019 Cinciripini, PM, et al. *JAMA Network Open.*

Statistical Analysis:

Supplementary Comparisons

In addition to the 3 major comparisons described in the main paper, we carried out supplementary comparisons to assess the impact on abstinence of: a) either having a smoking related (SMCa+) or unrelated (SMCa-) current cancer, and b) of being a cancer survivor (S). The no cancer history group (CaHx-) served as the reference.

Bivariate Analysis

We conducted simple bivariate comparisons of demographics and baseline variables shown in eTable 1 for the major cancer groupings used in the supplementary comparisons (CaHx-, SMCa+, SMCa-, S) using Chi-square for categorical comparisons and the *t*-test for continuous (*P* values are 2-sided).

Regression at Discrete Time Points

To provide covariate-adjusted risk ratios and associated standard errors we used the modified Poisson regression model[17–20] with a sandwich variance estimator to evaluate differences in abstinence for the comparisons of SMCa+ and SMCa- vs. CaHX-, and S vs. CaHx- using Stata version 14[21]. Separate models were evaluated for each comparison at each of the three time points (3, 6, and 9 months after consultation), that were both unadjusted and adjusted for the demographic and baseline covariates in Table S1. Usually, logistic regression is recommended when the probability of the event of interest is low and the baseline risks for subgroups are constant. In that case, the odds ratio (OR) and risk ratio (RR) converge[22]. However, when the probability of the outcome is not rare, and the baseline risks are not constant (as it is the case in our study), the interpretation of the OR as a RR leads to exaggeration of the actual probabilities. In addition, obtaining RRs from logistic regression is not straightforward and can be extremely tedious[23]. Also, there is a consensus to report RRs instead of ORs in clinical studies, because they represent consistent estimates of the actual risk[24], with valid confidence intervals that are easier to interpret than ORs[25]. For these reasons we chose the modified Poisson regression model over logistic regression to estimate RR point estimates with valid confidence limits. This method has been proposed as a superior alternative to logistic regression when estimating RRs[26–9], and has been extensively used in the analyses and publication of clinical studies[18,20,19].

Longitudinal Analysis

We used multivariate mixed effects modified Poisson regression models[18] to estimate the effects of longitudinal abstinence (over time) for both the major comparisons described in the main manuscript: CaHx+ vs. CaHx-; CaHx- vs each current cancer site; and the supplementary comparisons of SMCa+ and SMCa- vs. CaHx- ; and CaHx- vs. S. All models were both unadjusted and adjusted for covariates noted in eTable 1.

© 2019 Cinciripini, PM, et al. *JAMA Network Open.*

Statistical Software and Reporting

_All models were estimated in Stata statistical software version 14[21] using the "poisson" routine for the time-specific models and "xtpoisson" for the longitudinal mixed effects model modified[20,19] to estimate relative risks . For all comparisons in both sets of analyses, we estimated and report relative risks (RR) of abstinence with their corresponding 95% CIs and two-tailed P values. . Bonferroni corrections for multiple comparisons were applied by dividing .05/number comparisons X number of time points, within each model tested.

Supplementary Details for Multiple Imputation Approach

To implement the multiple imputation, we created 10 imputed datasets using Stata's "mi impute chained" command. This method uses a sequential regression multivariate imputation approach [27,28] to impute one variable at a time conditioning on all other variables, and using a Gibbs-like algorithm to obtain imputed values by simulating from the posterior predictive distribution. In addition, all imputations were performed separately for each cancer site category and were subsequently combined. Results from the multiple imputation datasets were analyzed and combined using Stata's "mi estimate" command. . In this study we evaluated abstinence rates using three different scenarios of missingness: a) Intention to Treat (ITT), b) Respondent Only (RO), and, c) Multiple Imputation (MI). This strategy of evaluating abstinence is suggested by Hedeker et al.[29] where they state that "it is important to examine results under a range of plausible values for the association of missing and smoking stratified by past smoking behavior" (p.1572). Although we did not stratify by past smoking behavior, we used past smoking behavior to predict missing smoking status and stratified by cancer group. In addition, our MI approach to imputation of missing smoking status is also recommended by Hedeker et al.[29] who state that "Our approach advocates the use of multiple imputation, because individual, sampling and imputation variation can be accounted for." (p. 1972). Although, all three missing methods we used provided comparable estimates of abstinence, with ITT being the most conservative, we believe that the MI method is more reliable since it utilizes information from individuals on demographics, psychosocial, and smoking-related characteristics to predict smoking status. In addition, our imputation accounted for cancer group membership by multiply imputing abstinence nested within cancer group. Acknowledging the fact that when missingness is substantial, no imputation method can reliably recover the true abstinence rates[30], we present in our manuscript all abstinence rates by follow-up time point and by imputation method (Figure 2 in manuscript). Given the relatively large number of covariates we included in the imputation model, and by nesting each imputation model within each cancer group, we believe that the MAR assumption given the covariates is plausible, and the multiple imputed estimates are the least biased from the three methods. This method of nested multiple imputation has been shown to produce reliable estimates and standard errors with nonignorable missing smoking data[31].

© 2019 Cinciripini, PM, et al. *JAMA Network Open.*

Response Rates

The response rates of the study sample (N=3245) for assessing abstinence were quite high for a clinical sample: 92%, 87%, and 83% for 3-, 6-, and 9-month time points for the participants who had a cancer diagnosis, and 91%, 84%, and 77% for the 3-, 6-, and 9-month time points for the participants without a cancer diagnosis. Overall, data on abstinence were missing only for 7.8% of the observations at the 3 month time point, 13.6% at the 6-month time point and 18.5% at the 9-month time point. The baseline covariates with missing data were FTCD (nicotine dependence) at 12%, years smoke at 9% and the PHQ (presence/absence of psychiatric comorbidities) at 9%. Missingness in baseline covariates was due to nonresponse on specific questionnaire items.

© 2019 Cinciripini, PM, et al. *JAMA Network Open.*

eResults. Demographics and Models

Demographics

As shown in eTable 1, compared with no cancer patients (CaHx-), cancer patients (CaHx+, SMCa+, SMCa-) were more likely to be older, male, white, and to smoke more (CPD), be more nicotine dependent (higher FTCD),); and to have smoked longer. Those with a cancer history or smoking related (CaHx+, SMCa+) also had a higher risk of having depression on the PHQ. Survivors (S) were older, White, smoked longer, and had a higher risk of anxiety on the PHQ. The smoking related cancer group was slightly more likely to be prescribed smoking cessation medication (96% vs. 94%).

Smoking Related Cancer and ITT and Longitudinal Models

No significant differences in abstinence rates were found in the comparisons of no cancer history (CaHx-) smoking-related (SMCa+) or non-smoking-related (SMCa-) cancer vs CaHx-, or survivors (S) at any the 3-, 6-, and 9-month follow-ups, nor in the longitudinal models (eTables 5-8). While nominally significant differences were noted in comparisons between SMCa+ and SMCa- groups (see eTables 5 and 6) in the multiply imputed sample, these effects did not survive correction for multiple comparisons ($p<.016$).

The ITT and respondent-only results were consistent with the multiply imputed results and showed no significant differences for these same comparisons (see supplement eTables 13-16) that survived correction for multiple comparisons ($p<.016$). Nominal differences were noted for the 9-month comparison of SMCa+ vs. CaHx- (eTable 13) in both ITT unadjusted and adjusted models and respondent only samples (eTable 14), but none survived correction for multiple comparisons ($p<.006$).

No abstinence differences were noted between cancer survivors (S) and the CaHx- group in either the multiply imputed (eFigure 3) model or the time specific (see eTables 20 and 21) or longitudinal models for the ITT or respondent only samples (eTables 22 and 23).

© 2019 Cinciripini, PM, et al. *JAMA Network Open.*

eTable 1. Baseline Characteristics of Patients (N = 3245)[a]					
**Characteristic**	**No Cancer History (CaHx-) (n=593)**	**Cancer History (CaHx+) (n=2652)**	**Survivor (S) (n=309)**	**Smoking Related Cancer (SMCa+) (n=1251)**	**Not-Smoking Related Cancer (SMCa-) (n=1092)**
**Age, mean (SD), y**	48.3 (12.3)	55.3 (10.8)	55.2 (11)	57 (10.3)	53.5 (11)
**P-value***		< 0.001	< 0.001	< 0.001	< 0.001
**Male, No. (%)**	237 (40)	1351 (50.9)	123 (39.8)	729 (58.3)	499 (45.7)
**P-value**		< 0.001	0.947	< 0.001	0.026
**Race, No. (%)**					
**Black**	71 (12)	251 (9.5)	43 (13.9)	101 (8.1)	107 (8.1)
**Hispanic**	29 (4.9)	143 (5.4)	16 (5.1)	58 (4.6)	69 (6.3)
**Other**	172 (29)	81 (3)	8 (2.6)	37 (3)	36 (3.3)
**White**	321 (54.1)	2177 (82.1)	242 (78.3)	1055 (84.1)	880 (80.6)
**P-value**		< 0.001	< 0.001	< 0.001	< 0.001
**Psychiatric Comorbidities on PHQ**					
**No**	318 (56)	1239 (52)	171 (57)	574 (51.4)	494 (51.1)
**Yes**	249 (44)	1144 (48)	129 (43)	542 (48.6)	473 (48.9)
**P-value**		0.079	0.818	0.065	0.051
**Anxiety**					
**No**	433 (76)	1796 (74)	252 (84)	824 (72)	721 (73)
**Yes**	138 (24)	635 (26)	49 (16)	318 (28)	268 (27)
**P-value**		0.337	0.007	0.109	0.211
**Alcohol**					
**No**	511 (89)	2201 (91)	274 (91)	1049 (92)	879 (89)
**Yes**	60 (11)	230 (9)	27 (9)	93 (8)	110 (11)
**P-value**		0.446	0.466	0.103	0.716
**Depression**					
**No**	429 (75)	1716 (71)	218 (72)	798 (70)	701 (71)
**Yes**	142(25)	717 (29)	83(28)	345 (30)	289 (29)
**P-value**		0.029	0.393	0.023	0.069
**Smoking Cessation Medication, No. (%)**					
**No**	23 (3.9)	156 (5.8)	20 (6.5)	82 (6.5)	54 (5.9)
**Yes**	570 (96.1)	2496 (94.2)	289 (93.5)	1169 (93.5)	1038 (95.1)
**P-value**		0.053	0.084	0.021	0.322
**FTCD (Nicotine Dependence), mean (SD)**	4.1 (2.2)	4.5 (2.2)	4.2 (2.3)	4.7 (2.2)	4.4 (2.2)
**P-value**		< 0.001	0.533	< 0.001	0.005
**CPD, mean (SD)**	16 (9.1)	17.4 (11)	15.7 (9.8)	17.8 (11.4)	17.4 (10.9)
**P-value**		0.006	0.586	0.001	.010
**Years Smoked, mean (SD)**	27 (13)	34.5 (12.8)	32.1 (13.9)	37 (12.4)	32.5 (12.5)
**P-value**		< 0.001	< 0.001	< 0.001	< 0.001

*all p-values are based on 2-tail t-test for continuous and chi-square test for categorical comparisons of a cancer group (cancer history, current cancer, survivor, smoking related and not-smoking related) vs. the no cancer history group.

a= Cancer History patients (CaHX+)=those with current malignancies or Survivors (S) with a previous but not current malignancy; No Cancer History (CaHx-)=employees and screening patients; Current Cancer Patients=those with (SMCa+) and without (SMCa-) smoking-related current malignancies.. Smoking-related cancers: Lung, head & neck, esophegal, colorectal, anal, appendix, extrahepatic bile duct, ileum, intrahepatic bile duct, liver pancreas, small intestine, stomach, cervical, labia, ovary, ureter, uterus, vagina, vulva, acute myeloid leukemia

© 2019 Cinciripini, PM, et al. *JAMA Network Open.*

eTable 2. Overall Abstinence (N = 3245)[a]						
	No Cancer History (CaHx-) (n=593)	Cancer History (CaHx+) (n=2652)	Survivor (S) (n=309)	Smoking Related Cancer (SMCa+) (n=1251)	Not-Smoking Related Cancer (SMCa-) (n=1092)	Total
**Multiply Imputed**	%	%	%	%	%	%
**3 Month**	43.8	45.5	45.7	47.4	43.2	45.1
**6 Month**	44.3	46	47.6	47.8	43.4	45.8
**9 Month**	41.5	44.6	43.2	46.5	42.7	43.7
**ITT**						
**3 Month**	39.5	41.4	42.1	42.5	39.9	41.1
**6 Month**	36.8	39.9	41.8	40.9	38.1	39.5
**9 Month**	31.5	36.5	37.2	37.5	35.3	35.6
**Respondent Only***	% (total n)	% (total n)	% (total n)	% (total n)	% (total n)	
**3 Month**	43.3 (540)	44.8 (2452)	45.3 (287)	46.4 (1146)	42.8 (1019)	44.5 (2992)
**6 Month**	43.9 (496)	45.8 (2306)	47.1 (274)	47.6 (1075)	43.5 (957)	45.6 (2802)
**9 Month**	41.2 (454)	44.3 (2189)	42.6 (270)	46.1 (1019)	42.8 (900)	43.7 (2643)
[a] Cancer History patients (CaHX+)=those with current malignancies or Survivors (S) with a previous but not current malignancy; No Cancer History (CaHx-)=employees and screening patients; Current Cancer Patients=those with (SMCa+) and without (SMCa-) smoking-related current malignancies. *For Respondent Only the total n is specific to each time point due to missing.						

© 2019 Cinciripini, PM, et al. *JAMA Network Open.*

eTable 3. Unadjusted and adjusted multiply imputed results for Cancer History (CaHx+) vs. No -cancer History (CaHx-) categories for each time point. Nominal p-values shown; multiple correction p-value over 3 time points=.016.

	3-Month			6-Month			9-Month		
	RR	95% CI	p-value	RR	95% CI	p-value	RR	95% CI	p-value
	Unadjusted								
**CaHx+**	1.03	0.93 - 1.14	0.57	1.03	0.93 - 1.15	0.53	1.08	0.96 - 1.21	0.20
	Adjusted								
**CaHx+**	1.03	0.93 - 1.16	0.55	1.05	0.94 - 1.18	0.38	1.10	0.97 - 1.26	0.140
**Age**	1.01**	1.00 - 1.01	0.003	1.01*	1.00 - 1.01	0.013	1.01**	1.00 - 1.01	0.002
**Gender**	1.13**	1.05 - 1.22	0.002	1.12**	1.04 - 1.22	0.0040	1.13**	1.04 - 1.23	0.004
**Race (ref = White)**									
**Black**	0.78**	0.67 - 0.91	0.0013	0.82**	0.71 - 0.95	0.0089	0.84*	0.72 - 0.98	0.023
**Hispanic**	0.91	0.76 - 1.09	0.30	0.98	0.82 - 1.17	0.83	1.04	0.89 - 1.22	0.60
**Other**	0.96	0.81 - 1.12	0.58	1.00	0.84 - 1.19	0.99	1.04	0.86 - 1.25	0.71
**Comorbidities**	0.88**	0.81 - 0.96	0.0037	0.91*	0.82 - 0.99	0.038	0.95	0.86 - 1.05	0.28
**Medication**	1.05	0.88 - 1.24	0.60	1.07	0.90 - 1.29	0.44	0.95	0.77 - 1.17	0.64
**CPD**	1.00	0.99 - 1.00	0.50	1.00	0.99 - 1.00	0.21	1.00	0.99 - 1.00	0.37
**Nicotine Dependence**	0.93***	0.90 - 0.95	< 0.001	0.94***	0.91 - 0.96	< 0.001	0.94***	0.92 - 0.97	< 0.001
**Years Smoked**	0.99**	0.99 - 1.00	0.0060	0.99**	0.99 - 1.00	0.0071	0.99***	0.99 - 1.00	< 0.001

© 2019 Cinciripini, PM, et al. *JAMA Network Open.*

eTable 4. Unadjusted and adjusted multiply imputed results for Cancer History (CaHx+) vs. No -cancer History (CaHx-) categories in longitudinal analyses

	Unadjusted			Adjusted		
	**RR**	**95% CI**	**p-value**	**RR**	**95% CI**	**p-value**
**CaHx+**	1.05	0.95 - 1.15	0.34	1.06	0.95 - 1.18	0.27
**Age**				1.01***	1.00 - 1.01	< 0.001
**Gender**				1.13***	1.05 - 1.21	< 0.001
**Race (ref = White)**						
**Black**				0.82**	0.72 - 0.93	0.002
**Hispanic**				0.98	0.84 - 1.14	0.76
**Other**				0.99	0.85 - 1.16	0.91
**Comorbidities**				0.91*	0.84 - 0.98	0.019
**Medication**				1.03	0.87 - 1.21	0.76
**CPD**				1.00	0.99 - 1.00	0.31
**Nicotine Dependence**				0.93***	0.91 - 0.96	< 0.001
**Years Smoked**				0.99***	0.99 - 1.00	< 0.001

© 2019 Cinciripini, PM, et al. *JAMA Network Open.*

eTable 5. Unadjusted and adjusted multiply-imputed results for smoking-related (SMCa+) and non-smoking related cancer (SMCa-) vs. no cancer history (CaHx-) and vs. each other for each time point. Nominal p-values shown; multiple correction p-value 3 comparisons over 3 time points=.006.

	3-Month			6-Month			9-Month		
	RR	95% CI	p-value	RR	95% CI	p-value	RR	95% CI	p-value
	Unadjusted								
**SMCa- vs. CaHx-**	0.98	0.87 - 1.10	0.75	0.98	0.87 - 1.11	0.76	1.04	0.92 - 1.19	0.52
**SMCa+ vs. CaHx-**	1.07	0.96 - 1.20	0.23	1.07	0.95 - 1.20	0.25	1.12	0.99 - 1.26	0.082
**SMCa+ SMCa-**	1.09	1.00 - 1.20	0.060	1.09	0.99 - 1.20	0.075	1.07	0.97 - 1.18	0.19
	Adjusted								
**SMCa- vs. CaHx-**	0.99	0.88 - 1.12	0.89	1.00	0.88 - 1.14	0.99	1.07	0.93 - 1.23	0.36
**SMCa+ vs. CaHx-**	1.09	0.96 - 1.23	0.17	1.09	0.96 - 1.24	0.16	1.15	1.00 - 1.33	0.055
**SMCa+ SMCa-**	1.10*	1.00 - 1.20	0.045	1.10	1.00 - 1.21	0.061	1.08	0.97 - 1.19	0.15
**Age**	1.01*	1.00 - 1.01	0.047	1.01	1.00 - 1.01	0.063	1.01*	1.00 - 1.01	0.012
**Gender**	1.12**	1.03 - 1.22	0.0063	1.12**	1.03 - 1.22	0.0078	1.12*	1.02 - 1.22	0.014
**Race (ref = White)**									
**Black**	0.81**	0.69 - 0.95	0.0087	0.83*	0.71 - 0.97	0.020	0.83*	0.70 - 0.98	0.028
**Hispanic**	0.93	0.78 - 1.11	0.40	0.98	0.82 - 1.18	0.83	1.02	0.86 - 1.22	0.79
**Other**	0.95	0.80 - 1.13	0.56	1.00	0.83 - 1.20	0.98	1.03	0.85 - 1.24	0.79
**Comorbidities**	0.89*	0.81 - 0.97	0.012	0.91*	0.82 - 1.00	0.049	0.93	0.84 - 1.03	0.17
**Medication**	1.05	0.88 - 1.27	0.58	1.10	0.90 - 1.34	0.37	0.97	0.77 - 1.22	0.79
**CPD**	1.00	0.99 - 1.00	0.62	1.00	0.99 - 1.00	0.45	1.00	0.99 - 1.00	0.50
**Nicotine Dependence**	0.92***	0.90 - 0.95	< 0.001	0.93***	0.90 - 0.95	< 0.001	0.94***	0.91 - 0.97	< 0.001
**Years Smoked**	1.00	0.99 - 1.00	0.057	1.00	0.99 - 1.00	0.080	0.99**	0.99 - 1.00	0.0050

© 2019 Cinciripini, PM, et al. *JAMA Network Open.*

eTable 6. Unadjusted and adjusted multiply-imputed results for smoking-related (SMCa+) and non-smoking related (SMCa-) cancer vs. no cancer history (CaHx-) and vs. each other for longitudinal analyses. Nominal p-values shown; multiple correction p-value for 3 comparisons=.016.

	Unadjusted				Adjusted		
	**RR**	**95% CI**	**p-value**		**RR**	**95% CI**	**p-value**
**SMCa- vs. CaHx-**	1.00	0.90 - 1.11	0.99		1.02	0.90 - 1.14	0.80
**SMCa+ vs. CaHx-**	1.08	0.98 - 1.20	0.12		1.11	0.99 - 1.25	0.070
**SMCa+ SMCa-**	1.08	1.00 - 1.17	0.053		1.09*	1.01 - 1.19	0.034
**Age**					1.01**	1.00 - 1.01	0.009
**Gender**					1.12**	1.04 - 1.21	0.002
**Race (ref = White)**							
**Black**					0.83**	0.72 - 0.95	0.007
**Hispanic**					0.98	0.83 - 1.15	0.76
**Other**					0.99	0.84 - 1.16	0.87
**Comorbidities**					0.91*	0.83 - 0.98	0.021
**Medication**					1.04	0.87 - 1.25	0.65
**CPD**					1.00	0.99 - 1.00	0.49
**Nicotine Dependence**					0.93***	0.91 - 0.95	< 0.001
**Years Smoked**					0.99*	0.99 - 1.00	0.010

© 2019 Cinciripini, PM, et al. *JAMA Network Open.*

eTable 7. Unadjusted and adjusted multiply imputed results for cancer survivor (S) vs. No Cancer history (CaHx-) and current cancer-all sites (CCa) for each time point. Nominal p-values shown; multiple correction p-value for 2 comparisons over 3 time points=.01.

	3-Month			6-Month			9-Month		
	**RR**	**95% CI**	**p-value**	**RR**	**95% CI**	**p-value**	**RR**	**95% CI**	**p-value**
	**Unadjusted**								
**S vs, CaHx-**	1.04	0.89 - 1.21	0.65	1.07	0.91 - 1.25	0.41	1.05	0.89 - 1.25	0.55
**S vs CCa**	1.01	0.88 - 1.15	0.91	1.04	0.91 - 1.19	0.57	0.97	0.84 - 1.13	0.73
	**Adjusted**								
**S vs, CaHx-**	1.01	0.86 - 1.19	0.88	1.06	0.90 - 1.24	0.50	1.05	0.88 - 1.25	0.61
**S vs CCa**	0.97	0.85 - 1.11	0.70	1.01	0.88 - 1.15	0.94	0.94	0.82 - 1.09	0.43
**Age**	1.01**	1.00 - 1.01	0.0034	1.01*	1.00 - 1.01	0.014	1.01**	1.00 - 1.01	0.0018
**Gender**	1.13**	1.05 - 1.22	0.0022	1.12**	1.04 - 1.22	0.0039	1.13**	1.04 - 1.22	0.0051
**Race (ref = White)**									
**Black**	0.78**	0.67 - 0.91	0.0013	0.82**	0.71 - 0.95	0.008	0.84*	0.72 - 0.98	0.024
**Hispanic**	0.91	0.76 - 1.09	0.30	0.98	0.82 - 1.17	0.83	1.04	0.89 - 1.22	0.60
**Other**	0.96	0.81 - 1.12	0.59	1.00	0.84 - 1.19	0.99	1.03	0.86 - 1.25	0.72
**Comorbidities**	0.88**	0.81 - 0.96	0.0036	0.91*	0.82 - 0.99	0.039	0.95	0.86 - 1.04	0.27
**Medication**	1.05	0.88 - 1.24	0.60	1.07	0.90 - 1.29	0.44	0.95	0.77 - 1.17	0.64
**CPD**	1.00	0.99 - 1.00	0.50	1.00	0.99 - 1.00	0.21	1.00	0.99 - 1.00	0.36
**Nicotine Dependence**	0.93***	0.90 - 0.95	< 0.001	0.94***	0.91 - 0.96	< 0.001	0.94***	0.92 - 0.97	< 0.001
**Years Smoked**	0.99**	0.99 - 1.00	0.0059	0.99**	0.99 - 1.00	0.0079	0.99***	0.99 - 1.00	< 0.001

© 2019 Cinciripini, PM, et al. *JAMA Network Open.*

eTable 8. Unadjusted and adjusted multiply imputed results for cancer survivor (S) vs. No Cancer history (CaHx-) and current cancer-all sites (CCa) for longitudinal analyses. Nominal p-values shown; multiple correction p-value for 2 comparisons=.025.

	Unadjusted			Adjusted		
	**RR**	**95% CI**	**p-value**	**RR**	**95% CI**	**p-value**
**S vs, CaHx-**	1.05	0.91 - 1.21	0.47	1.04	0.89 - 1.20	0.64
**S vs CCa**	1.01	0.89 - 1.13	0.90	0.97	0.86 - 1.10	0.66
**Age**				1.01***	1.00 - 1.01	< 0.001
**Gender**				1.13***	1.05 - 1.21	< 0.001
**Race (ref = White)**						
**Black**				0.82**	0.72 - 0.93	0.0024
**Hispanic**				0.98	0.84 - 1.14	0.76
**Other**				0.99	0.85 - 1.16	0.91
**Comorbidities**				0.91*	0.84 - 0.98	0.019
**Medication**				1.03	0.87 - 1.21	0.76
**CPD**				1.00	0.99 - 1.00	0.31
**Nicotine Dependence**				0.93***	0.91 - 0.96	< 0.001
**Years Smoked**				0.99***	0.99 - 1.00	< 0.001

© 2019 Cinciripini, PM, et al. *JAMA Network Open.*

eTable 9 Unadjusted and adjusted ITT results for Cancer History (CaHx+) vs. No -cancer History (CaHx-) categories for each time point. Nominal p-values shown; multiple correction p-value over 3 time points=.016.

	3-Month			6-Month			9-Month		
	RR	95% CI	p-value	RR	95% CI	p-value	RR	95% CI	p-value
	Unadjusted								
**CaHx+**	1.05	0.94 - 1.17	0.39	1.08	0.97 - 1.22	0.17	1.16*	1.02 - 1.32	0.025
	Adjusted								
**CaHx+**	1.05	0.93 - 1.19	0.40	1.10	0.96 - 1.25	0.16	1.20*	1.04 - 1.39	0.014
**Age**	1.01**	1.00 - 1.01	0.0018	1.01**	1.00 - 1.01	0.0013	1.01**	1.00 - 1.01	0.004
**Gender**	1.14**	1.04 - 1.24	0.0045	1.11*	1.01 - 1.22	0.024	1.11*	1.01 - 1.23	0.033
**Race (ref = White)**									
**Black**	0.80**	0.68 - 0.94	0.0070	0.85*	0.72 - 1.00	0.047	0.83*	0.70 - 0.99	0.042
**Hispanic**	0.93	0.77 - 1.13	0.46	0.97	0.80 - 1.18	0.78	1.04	0.86 - 1.27	0.67
**Other**	0.88	0.74 - 1.06	0.18	0.93	0.77 - 1.12	0.46	0.98	0.80 - 1.20	0.85
**Comorbidities**	0.87**	0.80 - 0.95	0.0021	0.89*	0.82 - 0.98	0.017	0.95	0.86 - 1.05	0.33
**Medication**	1.17	0.95 - 1.44	0.14	1.31*	1.04 - 1.65	0.024	1.12	0.90 - 1.40	0.31
**CPD**	1.00	0.99 - 1.01	0.86	1.00	0.99 - 1.01	0.74	1.00	0.99 - 1.00	0.57
**Nicotine Dependence**	0.93***	0.90 - 0.95	< 0.001	0.93***	0.90 - 0.95	< 0.001	0.93***	0.90 - 0.96	< 0.001
**Years Smoked**	0.99***	0.99 - 1.00	< 0.001	0.99**	0.99 - 1.00	0.0049	0.99**	0.99 - 1.00	0.0098

© 2019 Cinciripini, PM, et al. *JAMA Network Open.*

eTable10. Unadjusted and adjusted respondent-only results for Cancer History (CaHx+) vs. No -cancer History (CaHx-) categories for each time point. Nominal p-values shown; multiple correction p-value over 3 time points=.016.

	3-Month			6-Month			9-Month		
	**RR**	**95% CI**	**p-value**	**RR**	**95% CI**	**p-value**	**RR**	**95% CI**	**p-value**
	**Unadjusted**								
**CaHx+**	1.03	0.93 - 1.15	0.54	1.04	0.94 - 1.16	0.45	1.07	0.95 - 1.21	0.24
	**Adjusted**								
**CaHx+**	1.04	0.92 - 1.17	0.52	1.07	0.95 - 1.21	0.27	1.14	1.00 - 1.31	0.056
**Age**	1.01**	1.00 - 1.01	0.0050	1.01**	1.00 - 1.01	0.0097	1.01*	1.00 - 1.01	0.030
**Gender**	1.16***	1.07 - 1.27	< 0.001	1.14**	1.05 - 1.25	0.0026	1.15**	1.05 - 1.26	0.0027
**Race (ref = White)**									
**Black**	0.78**	0.67 - 0.92	0.0025	0.84*	0.72 - 0.98	0.028	0.83*	0.70 - 0.98	0.030
**Hispanic**	0.97	0.81 - 1.17	0.77	1.00	0.84 - 1.20	0.99	1.07	0.89 - 1.28	0.48
**Other**	0.92	0.77 - 1.10	0.36	0.97	0.81 - 1.15	0.72	1.03	0.86 - 1.24	0.75
**Comorbidities**	0.88**	0.81 - 0.96	0.0032	0.90*	0.83 - 0.99	0.022	0.96	0.88 - 1.05	0.38
**Medication**	1.06	0.87 - 1.29	0.55	1.17	0.94 - 1.46	0.15	0.97	0.80 - 1.18	0.77
**CPD**	1.00	0.99 - 1.00	0.78	1.00	0.99 - 1.00	0.49	1.00	0.99 - 1.00	0.37
**Nicotine Dependence**	0.93***	0.91 - 0.95	< 0.001	0.93***	0.91 - 0.96	< 0.001	0.94***	0.92 - 0.97	< 0.001
**Years Smoked**	0.99***	0.99 - 1.00	< 0.001	0.99**	0.99 - 1.00	0.0045	0.99**	0.99 - 1.00	0.0029

© 2019 Cinciripini, PM, et al. *JAMA Network Open.*

eTable 11. Unadjusted and adjusted ITT results for Cancer History (CaHx+) vs. No -cancer History (CaHx-) categories for the longitudinal analysis

	Unadjusted			Adjusted		
	**RR**	**95% CI**	**p-value**	**RR**	**95% CI**	**p-value**
**CaHx+**	1.09	0.99 - 1.21	0.092	1.11	0.98 - 1.25	0.09
**Age**				1.01***	1.00 - 1.01	< 0.001
**Gender**				1.13**	1.04 - 1.22	0.0048
**Race (ref = White)**						
**Black**				0.83*	0.72 - 0.96	0.01
**Hispanic**				0.98	0.82 - 1.17	0.83
**Other**				0.92	0.77 - 1.09	0.33
**Comorbidities**				0.90*	0.83 - 0.98	0.013
**Medication**				1.22	0.99 - 1.49	0.056
**CPD**				1	0.99 - 1.00	0.74
**Nicotine Dependence**				0.93***	0.90 - 0.95	< 0.001
**Years Smoked**				0.99***	0.99 - 1.00	< 0.001

© 2019 Cinciripini, PM, et al. *JAMA Network Open.*

eTable 12 Unadjusted and adjusted respondent-only results for Cancer History (CaHx+) vs. No -cancer History (CaHx-) categories for the longitudinal analysis.

	Unadjusted			Adjusted		
	**RR**	**95% CI**	**p-value**	**RR**	**95% CI**	**p-value**
**CaHx+**	1.05	0.95 - 1.16	0.33	1.08	0.96 - 1.21	0.19
**Age**				1.01**	1.00 - 1.01	0.002
**Gender**				1.15***	1.07 - 1.24	< 0.001
**Race (ref = White)**						
**Black**				0.82**	0.71 - 0.94	0.004
**Hispanic**				1.01	0.86 - 1.19	0.90
**Other**				0.96	0.82 - 1.13	0.65
**Comorbidities**				0.91*	0.84 - 0.98	0.017
**Medication**				1.07	0.89 - 1.29	0.47
**CPD**				1.00	0.99 - 1.00	0.50
**Nicotine Dependence**				0.93***	0.91 - 0.95	< 0.001
**Years Smoked**				0.99***	0.99 - 1.00	< 0.001

© 2019 Cinciripini, PM, et al. *JAMA Network Open.*

eTable 13. Unadjusted and adjusted ITT results for smoking-related (SMCa+) and non-smoking related (SMCa-) cancer vs. no cancer history (CaHx-) and vs. each other each time point. Nominal p-values shown; multiple correction p-value for the 3 comparisons over 3 time points=.006.

	3-Month			6-Month			9-Month		
	RR	95% CI	p-value	RR	95% CI	p-value	RR	95% CI	p-value
					Unadjusted				
SMCa- vs CaHx-	1.01	0.89 - 1.14	0.88	1.03	0.91 - 1.18	0.62	1.12	0.97 - 1.29	0.14
SMCa+ vs CaHx-	1.08	0.96 - 1.21	0.23	1.11	0.98 - 1.26	0.097	1.19*	1.03 - 1.36	0.015
SMCa+ vs. SMCa-	1.07	0.97 - 1.17	0.20	1.08	0.97 - 1.19	0.16	1.06	0.96 - 1.19	0.26
					Adjusted				
SMCa- vs CaHx-	1.02	0.89 - 1.17	0.74	1.05	0.91 - 1.21	0.52	1.17	1.00 - 1.37	0.052
SMCa+ vs CaHx-	1.11	0.97 - 1.27	0.14	1.14	0.99 - 1.32	0.064	1.25**	1.06 - 1.46	0.0065
SMCa+ vs. SMCa-	1.08	0.98 - 1.20	0.13	1.09	0.98 - 1.21	0.11	1.07	0.95 - 1.19	0.26
Age	1.01*	1.00 - 1.01	0.017	1.01**	1.00 - 1.02	0.0035	1.01*	1.00 - 1.02	0.013
Gender	1.13*	1.03 - 1.24	0.012	1.11*	1.00 - 1.22	0.040	1.10	0.99 - 1.22	0.082
Race (ref = White)									
Black	0.83*	0.70 - 0.98	0.032	0.88	0.74 - 1.04	0.13	0.83	0.68 - 1.01	0.060
Hispanic	0.97	0.80 - 1.18	0.78	1.01	0.82 - 1.23	0.94	1.07	0.88 - 1.32	0.49
Other	0.88	0.73 - 1.06	0.16	0.93	0.76 - 1.13	0.45	0.96	0.78 - 1.19	0.73
Comorbidities	0.88**	0.81 - 0.97	0.0095	0.90*	0.82 - 0.99	0.032	0.93	0.84 - 1.04	0.20
Medication	1.18	0.94 - 1.48	0.16	1.35*	1.04 - 1.75	0.024	1.14	0.89 - 1.46	0.30
CPD	1.00	0.99 - 1.01	0.97	1.00	0.99 - 1.01	0.80	1.00	0.99 - 1.01	0.77
Nicotine Dependence	0.92***	0.89 - 0.94	< 0.001	0.92***	0.89 - 0.94	< 0.001	0.93***	0.90 - 0.95	< 0.001
Years Smoked	0.99**	0.99 - 1.00	0.0044	0.99*	0.99 - 1.00	0.027	0.99*	0.99 - 1.00	0.032

© 2019 Cinciripini, PM, et al. *JAMA Network Open.*

eTable 14. Unadjusted and adjusted respondent-only results for smoking-related (SMCa+) and non-smoking related (SMCa-) cancer vs. no cancer history (CaHx-) and vs. each other for each time point. Nominal p-values shown; multiple correction p-value for the 3 comparisons over 3 time points=.006.

	3-Month			6-Month			9-Month		
	RR	95% CI	p-value	RR	95% CI	p-value	RR	95% CI	p-value
	Unadjusted								
**SMCa- vs CaHx-**	0.99	0.88 - 1.11	0.84	0.99	0.87 - 1.12	0.86	1.04	0.91 - 1.19	0.58
**SMCa+ vs CaHx-**	1.07	0.96 - 1.20	0.24	1.08	0.96 - 1.22	0.18	1.12	0.98 - 1.27	0.090
**SMCa+ vs. SMCa-**	1.08	0.99 - 1.19	0.090	1.10	1.00 - 1.21	0.061	1.08	0.97 - 1.19	0.15
	Adjusted								
**SMCa- vs CaHx-**	1.00	0.88 - 1.15	0.94	1.02	0.89 - 1.17	0.76	1.12	0.97 - 1.30	0.13
**SMCa+ vs CaHx-**	1.10	0.97 - 1.26	0.15	1.13	0.99 - 1.30	0.076	1.20*	1.04 - 1.40	0.014
**SMCa+ vs. SMCa-**	1.10	0.99 - 1.21	0.068	1.11*	1.00 - 1.22	0.048	1.08	0.97 - 1.20	0.18
**Age**	1.01	1.00 - 1.01	0.062	1.01*	1.00 - 1.01	0.032	1.01	1.00 - 1.01	0.082
**Gender**	1.15**	1.05 - 1.26	0.0021	1.14**	1.04 - 1.25	0.0059	1.14*	1.03 - 1.25	0.011
**Race (ref = White)**									
**Black**	0.81*	0.68 - 0.96	0.013	0.86	0.73 - 1.02	0.081	0.83*	0.69 - 1.00	0.046
**Hispanic**	0.98	0.81 - 1.19	0.86	1.00	0.83 - 1.21	0.97	1.06	0.88 - 1.28	0.52
**Other**	0.92	0.76 - 1.10	0.34	0.97	0.81 - 1.17	0.75	1.02	0.84 - 1.24	0.82
**Comorbidities**	0.89*	0.82 - 0.98	0.015	0.91*	0.83 - 0.99	0.036	0.94	0.85 - 1.03	0.20
**Medication**	1.08	0.87 - 1.34	0.48	1.23	0.96 - 1.57	0.096	1.00	0.80 - 1.24	0.97
**CPD**	1.00	0.99 - 1.01	0.87	1.00	0.99 - 1.01	0.82	1.00	0.99 - 1.00	0.45
**Nicotine Dependence**	0.92***	0.90 - 0.95	< 0.001	0.92***	0.90 - 0.95	< 0.001	0.94***	0.91 - 0.97	< 0.001
**Years Smoked**	0.99*	0.99 - 1.00	0.013	0.99*	0.99 - 1.00	0.041	0.99*	0.99 - 1.00	0.015

© 2019 Cinciripini, PM, et al. *JAMA Network Open.*

eTable 15. Unadjusted and adjusted ITT results for smoking-related (SMCa+) and non-smoking related (SMCa-) cancer vs. no cancer history (CaHx-) and vs. each other for longitudinal analyses. Nominal p-values shown; multiple correction p-value for 3 comparisons=.016.

	Unadjusted			Adjusted		
	**RR**	**95% CI**	**p-value**	**RR**	**95% CI**	**p-value**
**SMCa- vs CaHx-**	1.05	0.93 - 1.18	0.42	1.07	0.94 - 1.21	0.32
**SMCa+ vs CaHx-**	1.12*	1.00 - 1.25	0.046	1.16*	1.02 - 1.32	0.027
**SMCa+ vs. SMCa-**	1.07	0.98 - 1.17	0.14	1.09	0.99 - 1.20	0.090
**Age**				1.01**	1.00 - 1.01	0.002
**Gender**				1.12*	1.02 - 1.22	0.012
**Race (ref = White)**						
**Black**				0.85*	0.73 - 0.99	0.035
**Hispanic**				1.01	0.84 - 1.22	0.90
**Other**				0.91	0.76 - 1.08	0.29
**Comorbidities**				0.90*	0.83 - 0.98	0.018
**Medication**				1.23	0.99 - 1.54	0.061
**CPD**				1.00	0.99 - 1.01	0.99
**Nicotine Dependence**				0.92***	0.90 - 0.94	< 0.001
**Years Smoked**				0.99**	0.99 - 1.00	0.006

© 2019 Cinciripini, PM, et al. *JAMA Network Open.*

eTable 16. Unadjusted and adjusted respondent-only results for smoking-related vs non-smoking related categories for longitudinal analyses. Nominal p-values shown; multiple correction p-value for 3 comparisons=.016.

	Unadjusted			Adjusted		
	**RR**	**95% CI**	**p-value**	**RR**	**95% CI**	**p-value**
**SMCa- vs CaHx-**	1.00	0.90 - 1.12	0.95	1.04	0.92 - 1.17	0.53
**SMCa+ vs CaHx-**	1.09	0.98 - 1.21	0.11	1.14*	1.01 - 1.29	0.034
**SMCa+ vs. SMCa-**	1.08	1.00 - 1.18	0.057	1.10*	1.00 - 1.20	0.039
**Age**				1.01*	1.00 - 1.01	0.020
**Gender**				1.14**	1.05 - 1.24	0.001
**Race (ref = White)**						
**Black**				0.84*	0.72 - 0.97	0.017
**Hispanic**				1.01	0.85 - 1.20	0.87
**Other**				0.96	0.82 - 1.13	0.63
**Comorbidities**				0.91*	0.84 - 0.99	0.021
**Medication**				1.10	0.90 - 1.35	0.37
**CPD**				1.00	0.99 - 1.00	0.67
**Nicotine Dependence**				0.93***	0.91 - 0.95	< 0.001
**Years Smoked**				0.99**	0.99 - 1.00	0.007

© 2019 Cinciripini, PM, et al. *JAMA Network Open.*

eTable 17. Unadjusted and adjusted multiply-imputed results for cancer site categories vs. no cancer history (CaHx-). Nominal p-values shown; multiple correction p-value for 9 comparisons over 3 time points=.002

	3-Month			6-Month			9-Month		
	RR	95% CI	p-value	RR	95% CI	p-value	RR	95% CI	p-value
	Unadjusted								
**Breast**	0.95	0.80 - 1.13	0.55	1.03	0.87 - 1.22	0.7	1.18	1.00 - 1.40	0.055
**Colorectal & Other GI**	0.94	0.78 - 1.14	0.55	1.03	0.86 - 1.22	0.77	1.02	0.84 - 1.23	0.86
**H&N**	1.18*	1.03 - 1.35	0.019	1.19*	1.04 - 1.37	0.014	1.30***	1.12 - 1.49	< 0.001
**Lung**	1.12	0.97 - 1.30	0.11	1.09	0.93 - 1.26	0.28	1.12	0.95 - 1.32	0.17
**Lymphoma/Hematological**	1.01	0.86 - 1.18	0.89	0.92	0.77 - 1.10	0.37	0.98	0.81 - 1.18	0.82
**Melanoma & Other Skin**	0.96	0.77 - 1.20	0.72	0.96	0.77 - 1.21	0.75	1	0.78 - 1.28	1
**other**	0.95	0.80 - 1.13	0.57	1	0.84 - 1.19	1	1.02	0.85 - 1.22	0.84
**Other GU**	0.95	0.80 - 1.12	0.53	0.9	0.75 - 1.08	0.26	0.91	0.75 - 1.11	0.36
**Prostate**	1.08	0.87 - 1.35	0.49	0.99	0.78 - 1.27	0.96	1	0.78 - 1.29	0.99
	Adjusted								
**Breast**	1	0.84 - 1.20	1	1.11	0.93 - 1.33	0.26	1.28	1.07 - 1.54	0.008
**Colorectal & Other GI**	0.93	0.77 - 1.13	0.49	1.01	0.84 - 1.22	0.91	1.01	0.82 - 1.23	0.94
**H&N**	1.20*	1.04 - 1.39	0.015	1.21*	1.05 - 1.41	0.01	1.31**	1.11 - 1.55	0.001
**Lung**	1.15	0.99 - 1.35	0.074	1.11	0.95 - 1.30	0.19	1.17	0.97 - 1.41	0.099
**Lymphoma/Hematological**	1	0.85 - 1.18	1	0.91	0.76 - 1.09	0.32	0.97	0.80 - 1.18	0.76
**Melanoma & Other Skin**	0.98	0.79 - 1.22	0.86	0.99	0.79 - 1.24	0.92	1.03	0.80 - 1.32	0.83
**other**	0.99	0.83 - 1.18	0.91	1.04	0.87 - 1.24	0.65	1.06	0.88 - 1.29	0.52
**Other GU**	0.99	0.83 - 1.18	0.95	0.95	0.79 - 1.15	0.59	0.97	0.78 - 1.19	0.74
**Prostate**	0.99	0.79 - 1.25	0.96	0.91	0.70 - 1.17	0.45	0.92	0.70 - 1.20	0.54
**Age**	1.01	1.00 - 1.01	0.054	1.01	1.00 - 1.01	0.063	1.01*	1.00 - 1.01	0.013
**Gender**	1.12*	1.02 - 1.22	0.015	1.14**	1.04 - 1.25	0.004	1.16**	1.05 - 1.28	0.003
**Race (ref = White)**									
**Black**	0.81**	0.69 - 0.95	0.0081	0.83*	0.71 - 0.97	0.018	0.82*	0.70 - 0.97	0.021
**Hispanic**	0.93	0.78 - 1.12	0.45	0.99	0.83 - 1.18	0.91	1.03	0.87 - 1.22	0.71
**Other**	0.96	0.81 - 1.13	0.61	1	0.84 - 1.20	1	1.03	0.85 - 1.25	0.77
**Comorbidities**	0.89**	0.81 - 0.97	0.009	0.90*	0.81 - 0.99	0.031	0.92	0.83 - 1.02	0.11
**Medication**	1.07	0.89 - 1.29	0.49	1.12	0.91 - 1.37	0.27	0.99	0.79 - 1.26	0.96

© 2019 Cinciripini, PM, et al. *JAMA Network Open.*

	3-Month			6-Month			9-Month		
	**RR**	**95% CI**	**p-value**	**RR**	**95% CI**	**p-value**	**RR**	**95% CI**	**p-value**
**CPD**	1	0.99 - 1.00	0.65	1	0.99 - 1.00	0.48	1	0.99 - 1.00	0.53
**Nicotine Dependence**	0.92***	0.90 - 0.95	< 0.001	0.93***	0.90 - 0.95	< 0.001	0.94***	0.91 - 0.97	< 0.001
**Years Smoked**	1	0.99 - 1.00	0.05	1	0.99 - 1.00	0.071	0.99**	0.99 - 1.00	0.005

© 2019 Cinciripini, PM, et al. *JAMA Network Open.*

eTable 18. Unadjusted and adjusted ITT results for cancer site categories vs. no cancer history (CaHx-). Nominal p-values shown; multiple correction p-value for 9 comparisons over 3 time points=.002.

	3-Month			6-Month			9-Month		
	RR	95% CI	p-value	RR	95% CI	p-value	RR	95% CI	p-value
	Unadjusted								
Breast	0.99	0.83 - 1.19	0.92	1.09	0.91 - 1.31	0.33	1.24*	1.02 - 1.50	0.028
Colorectal & Other GI	0.95	0.78 - 1.16	0.62	1.06	0.87 - 1.29	0.57	1.09	0.88 - 1.36	0.43
H&N	1.17*	1.02 - 1.35	0.030	1.22**	1.05 - 1.42	0.009	1.34***	1.14 - 1.58	< 0.001
Lung	1.18*	1.01 - 1.37	0.035	1.18*	1.01 - 1.39	0.041	1.26*	1.05 - 1.50	0.011
Lymphoma/Hematological	1.06	0.89 - 1.25	0.51	0.98	0.81 - 1.18	0.82	1.06	0.87 - 1.30	0.54
Melanoma & Other Skin	1.01	0.81 - 1.26	0.93	0.97	0.76 - 1.24	0.81	1.06	0.82 - 1.38	0.64
other	0.96	0.80 - 1.14	0.62	1.06	0.89 - 1.27	0.49	1.14	0.94 - 1.39	0.19
Other GU	0.91	0.76 - 1.10	0.33	0.91	0.75 - 1.11	0.35	0.96	0.77 - 1.19	0.69
Prostate	1.08	0.85 - 1.37	0.51	1.06	0.82 - 1.37	0.64	0.98	0.72 - 1.33	0.90
	Adjusted								
Breast	1.05	0.87 - 1.27	0.62	1.14	0.94 - 1.39	0.18	1.31*	1.06 - 1.61	0.011
Colorectal & Other GI	0.93	0.75 - 1.16	0.52	1.05	0.85 - 1.30	0.65	1.08	0.85 - 1.37	0.52
H&N	1.18*	1.00 - 1.39	0.045	1.25*	1.05 - 1.48	0.011	1.35**	1.12 - 1.63	0.001
Lung	1.25*	1.05 - 1.48	0.011	1.22*	1.02 - 1.46	0.033	1.35**	1.11 - 1.65	0.003
Lymphoma/Hematological	1.04	0.86 - 1.25	0.68	1.01	0.83 - 1.23	0.90	1.14	0.93 - 1.41	0.21
Melanoma & Other Skin	1.04	0.82 - 1.31	0.76	0.98	0.76 - 1.26	0.87	1.13	0.87 - 1.47	0.36
other	1.00	0.83 - 1.22	0.98	1.08	0.89 - 1.32	0.44	1.20	0.97 - 1.48	0.099
Other GU	0.99	0.81 - 1.22	0.96	0.97	0.78 - 1.20	0.77	1.05	0.83 - 1.33	0.66
Prostate	1.02	0.79 - 1.31	0.88	0.96	0.73 - 1.27	0.79	0.92	0.67 - 1.27	0.60
Age	1.01*	1.00 - 1.01	0.023	1.01**	1.00 - 1.02	0.004	1.01**	1.00 - 1.02	0.009
Gender	1.13*	1.02 - 1.25	0.020	1.12*	1.01 - 1.25	0.032	1.13*	1.01 - 1.27	0.032
Race (ref = White)									
Black	0.82*	0.69 - 0.98	0.028	0.87	0.73 - 1.03	0.11	0.82	0.68 - 1.00	0.051
Hispanic	0.98	0.81 - 1.20	0.86	1.01	0.83 - 1.24	0.89	1.08	0.89 - 1.33	0.44
Other	0.88	0.73 - 1.06	0.19	0.93	0.76 - 1.12	0.44	0.96	0.78 - 1.19	0.73
Comorbidities	0.89*	0.80 - 0.99	0.031	0.89*	0.80 - 0.99	0.035	0.90	0.80 - 1.01	0.073

© 2019 Cinciripini, PM, et al. *JAMA Network Open.*

	3-Month			6-Month			9-Month		
	RR	95% CI	p-value	RR	95% CI	p-value	RR	95% CI	p-value
**Medication**	0.88**	0.80 - 0.97	0.0081	0.89*	0.81 - 0.98	0.020	0.93	0.84 - 1.03	0.15
**CPD**	1.19	0.94 - 1.49	0.14	1.38*	1.06 - 1.79	0.015	1.16	0.91 - 1.49	0.23
**Nicotine Dependence**	1.00	0.99 - 1.01	0.95	1.00	1.00 - 1.01	0.69	1.00	0.99 - 1.01	0.90
**Years Smoked**	0.92***	0.89 - 0.94	< 0.001	0.92***	0.89 - 0.94	< 0.001	0.93***	0.90 - 0.95	< 0.001

© 2019 Cinciripini, PM, et al. *JAMA Network Open.*

eTable 19. Unadjusted and adjusted respondent-only results for cancer site categories vs. no cancer history (CaHx-) for each time point. Nominal p-values shown; multiple correction p-value for 9 comparisons over 3 time points=.002.

	3-Month			6-Month			9-Month		
	RR	95% CI	p-value	RR	95% CI	p-value	RR	95% CI	p-value
	Unadjusted								
Breast	0.95	0.80 - 1.14	0.59	1.03	0.86 - 1.22	0.76	1.15	0.96 - 1.37	0.13
Colorectal & Other GI	0.94	0.78 - 1.14	0.55	1.04	0.86 - 1.25	0.71	1.01	0.82 - 1.24	0.94
H&N	1.19*	1.03 - 1.36	0.014	1.23**	1.07 - 1.41	0.004	1.33***	1.14 - 1.54	< 0.001
Lung	1.14	0.98 - 1.32	0.091	1.10	0.94 - 1.28	0.22	1.13	0.95 - 1.33	0.16
Lymphoma/Hematological	1.03	0.87 - 1.21	0.75	0.94	0.79 - 1.12	0.50	1.00	0.83 - 1.21	1.00
Melanoma & Other Skin	0.97	0.78 - 1.21	0.77	0.91	0.72 - 1.16	0.45	0.96	0.75 - 1.22	0.72
other	0.95	0.80 - 1.13	0.55	1.02	0.86 - 1.21	0.81	1.06	0.88 - 1.27	0.55
Other GU	0.92	0.77 - 1.10	0.35	0.89	0.73 - 1.07	0.21	0.90	0.73 - 1.10	0.29
Prostate	1.10	0.87 - 1.38	0.43	1.04	0.82 - 1.33	0.75	0.94	0.71 - 1.25	0.66
	Adjusted								
Breast	1.03	0.85 - 1.24	0.77	1.10	0.91 - 1.32	0.33	1.26*	1.04 - 1.53	0.020
Colorectal & Other GI	0.92	0.74 - 1.13	0.41	1.03	0.84 - 1.27	0.79	1.02	0.82 - 1.28	0.84
H&N	1.19*	1.01 - 1.39	0.037	1.25**	1.07 - 1.47	0.006	1.36***	1.15 - 1.61	< 0.001
Lung	1.22*	1.03 - 1.44	0.021	1.17	0.98 - 1.38	0.079	1.26*	1.05 - 1.52	0.014
Lymphoma/Hematological	1.01	0.84 - 1.20	0.95	0.98	0.81 - 1.18	0.85	1.09	0.90 - 1.32	0.39
Melanoma & Other Skin	1.00	0.80 - 1.26	0.98	0.95	0.75 - 1.22	0.71	1.07	0.83 - 1.37	0.61
other	0.99	0.82 - 1.19	0.90	1.05	0.87 - 1.26	0.63	1.13	0.92 - 1.38	0.24
Other GU	1.01	0.83 - 1.23	0.89	0.97	0.79 - 1.20	0.80	1.02	0.81 - 1.27	0.88
Prostate	1.03	0.81 - 1.31	0.79	0.98	0.76 - 1.28	0.91	0.94	0.69 - 1.27	0.67
Age	1.01	1.00 - 1.01	0.077	1.01*	1.00 - 1.01	0.035	1.01	1.00 - 1.01	0.074
Gender	1.15**	1.04 - 1.27	0.005	1.14**	1.03 - 1.27	0.009	1.16**	1.04 - 1.29	0.006
Race (ref = White)									
Black	0.80*	0.68 - 0.95	0.011	0.86	0.73 - 1.01	0.071	0.82*	0.69 - 0.99	0.038
Hispanic	0.99	0.82 - 1.20	0.94	1.01	0.84 - 1.22	0.92	1.08	0.89 - 1.29	0.44
Other	0.92	0.77 - 1.11	0.38	0.97	0.81 - 1.17	0.77	1.03	0.85 - 1.24	0.79
Comorbidities	0.89*	0.82 - 0.98	0.013	0.90*	0.82 - 0.99	0.023	0.93	0.84 - 1.02	0.13
Medication	1.09	0.88 - 1.35	0.43	1.26	0.99 - 1.61	0.059	1.03	0.83 - 1.29	0.77

© 2019 Cinciripini, PM, et al. *JAMA Network Open.*

	3-Month			6-Month			9-Month		
	RR	95% CI	p-value	RR	95% CI	p-value	RR	95% CI	p-value
**CPD**	1.00	0.99 - 1.01	0.91	1.00	0.99 - 1.01	0.86	1.00	0.99 - 1.00	0.53
**Nicotine Dependence**	0.92***	0.90 - 0.95	< 0.001	0.92***	0.90 - 0.95	< 0.001	0.94***	0.91 - 0.97	< 0.001
**Years Smoked**	0.99*	0.99 - 1.00	0.010	0.99*	0.99 - 1.00	0.042	0.99*	0.99 - 1.00	0.014

© 2019 Cinciripini, PM, et al. *JAMA Network Open.*

eTable 20. Unadjusted and adjusted ITT results for cancer survivor (S) vs. No cancer (CaHx-) and current cancer (CCa) for each time point. Nominal p-values shown; multiple correction p-value for 2 comparisons over 3 time points=.008.

	3-Month			6-Month			9-Month		
	RR	95% CI	p-value	RR	95% CI	p-value	RR	95% CI	p-value
	Unadjusted								
S vs. CaHx-	1.06	0.90 - 1.25	0.46	1.13	0.96 - 1.34	0.15	1.18	0.98 - 1.42	0.086
S vs. CCa	1.02	0.89 - 1.17	0.79	1.05	0.92 - 1.21	0.46	1.02	0.88 - 1.19	0.79
	Adjusted								
S vs. CaHx-	0.98	0.82 - 1.17	0.85	1.06	0.88 - 1.27	0.54	1.11	0.91 - 1.36	0.31
S vs. CCa	0.92	0.80 - 1.07	0.29	0.96	0.83 - 1.11	0.59	0.92	0.78 - 1.08	0.30
Age	1.01**	1.00 - 1.01	0.0015	1.01**	1.00 - 1.01	0.0012	1.01**	1.00 - 1.02	0.0033
Gender	1.13**	1.04 - 1.24	0.0055	1.11*	1.01 - 1.22	0.026	1.11*	1.01 - 1.23	0.038
Race (ref = White)									
Black	0.80**	0.68 - 0.94	0.0075	0.85*	0.72 - 1.00	0.048	0.83*	0.70 - 1.00	0.044
Hispanic	0.93	0.77 - 1.13	0.46	0.97	0.80 - 1.18	0.77	1.04	0.86 - 1.27	0.68
Other	0.88	0.74 - 1.06	0.18	0.93	0.77 - 1.12	0.45	0.98	0.80 - 1.20	0.84
Comorbidities	0.87**	0.79 - 0.95	0.0018	0.89*	0.82 - 0.98	0.016	0.95	0.86 - 1.05	0.31
Medication	1.17	0.95 - 1.44	0.15	1.31*	1.03 - 1.65	0.025	1.12	0.89 - 1.40	0.33
CPD	1.00	0.99 - 1.01	0.85	1.00	0.99 - 1.01	0.73	1.00	0.99 - 1.00	0.56
Nicotine Dependence	0.92***	0.90 - 0.95	< 0.001	0.93***	0.90 - 0.95	< 0.001	0.93***	0.90 - 0.96	< 0.001
Years Smoked	0.99***	0.99 - 1.00	< 0.001	0.99**	0.99 - 1.00	0.0045	0.99**	0.99 - 1.00	0.0078

© 2019 Cinciripini, PM, et al. *JAMA Network Open.*

eTable 21. Unadjusted and adjusted respondent only results for cancer survivor (S) vs. No cancer (CaHx-) and current cancer (CCa) for each time point. Nominal p-values shown; multiple correction p-value for 2 comparisons over 3 time points=.008.

	3-Month			6-Month			9-Month		
	RR	95% CI	p-value	RR	95% CI	p-value	RR	95% CI	p-value
	Unadjusted								
S vs. CaHx-	1.05	0.89 - 1.23	0.59	1.07	0.91 - 1.26	0.40	1.03	0.87 - 1.23	0.71
S vs. CCa	1.01	0.88 - 1.16	0.85	1.03	0.90 - 1.18	0.66	0.96	0.83 - 1.11	0.56
	Adjusted								
S vs. CaHx-	0.98	0.83 - 1.16	0.83	1.02	0.86 - 1.22	0.81	1.01	0.84 - 1.23	0.89
S vs. CCa	0.93	0.81 - 1.08	0.34	0.95	0.82 - 1.09	0.44	0.87	0.75 - 1.01	0.076
Age	1.01**	1.00 - 1.01	0.0044	1.01**	1.00 - 1.01	0.0089	1.01*	1.00 - 1.01	0.024
Gender	1.16***	1.06 - 1.26	< 0.001	1.14**	1.05 - 1.24	0.0030	1.15**	1.04 - 1.26	0.0038
Race (ref = White)									
Black	0.78**	0.67 - 0.92	0.0027	0.84*	0.72 - 0.98	0.029	0.83*	0.70 - 0.99	0.033
Hispanic	0.97	0.81 - 1.16	0.75	1.00	0.83 - 1.20	1.00	1.06	0.89 - 1.27	0.51
Other	0.92	0.77 - 1.10	0.36	0.97	0.81 - 1.15	0.72	1.03	0.86 - 1.24	0.74
Comorbidities	0.88**	0.81 - 0.96	0.0029	0.90*	0.83 - 0.98	0.021	0.96	0.87 - 1.05	0.34
Medication	1.06	0.87 - 1.29	0.56	1.17	0.94 - 1.46	0.15	0.97	0.79 - 1.18	0.74
CPD	1.00	0.99 - 1.00	0.77	1.00	0.99 - 1.00	0.48	1.00	0.99 - 1.00	0.35
Nicotine Dependence	0.93***	0.91 - 0.95	< 0.001	0.93***	0.91 - 0.96	< 0.001	0.94***	0.92 - 0.97	< 0.001
Years Smoked	0.99***	0.99 - 1.00	< 0.001	0.99**	0.99 - 1.00	0.0040	0.99**	0.99 - 1.00	0.0019

© 2019 Cinciripini, PM, et al. *JAMA Network Open.*

eTable 22. Unadjusted and adjusted ITT results for cancer survivor (S) vs. No cancer (CaHx-) and current cancer (CCa). Nominal p-values shown; multiple correction p-value for 2 comparisons=.025.

	Unadjusted			Adjusted		
	RR	95% CI	p-value	RR	95% CI	p-value
**S vs. CaHx-**	1.12	0.96 - 1.31	0.140	1.04	0.88 - 1.23	0.610
**S vs. CCa**	1.03	0.91 - 1.17	0.630	0.94	0.82 - 1.07	0.330
**Age**				1.01***	1.00 - 1.01	< 0.001
**Gender**				1.12**	1.03 - 1.22	0.0058
**Race (ref = White)**						
**Black**				0.83*	0.72 - 0.96	0.011
**Hispanic**				0.98	0.82 - 1.17	0.820
**Other**				0.92	0.77 - 1.09	0.320
**Comorbidities**				0.90*	0.83 - 0.98	0.012
**Medication**				1.22	0.99 - 1.49	0.060
**CPD**				1.00	0.99 - 1.00	0.730
**Nicotine Dependence**				0.93***	0.90 - 0.95	< 0.001
**Years Smoked**				0.99***	0.99 - 1.00	< 0.001

© 2019 Cinciripini, PM, et al. *JAMA Network Open.*

eTable 23. Unadjusted and adjusted respondent only results for cancer survivor (S) vs. No cancer (CaHx-) and current cancer (CCa). Nominal p-values shown; multiple correction p-value for 2 comparisons =.025.

	Unadjusted			Adjusted		
	**RR**	**95% CI**	**p-value**	**RR**	**95% CI**	**p-value**
**S vs. CaHx-**	1.05	0.91 - 1.22	0.49	1.09	0.97 - 1.22	0.13
**S vs. CCa**	1.00	0.89 - 1.13	0.96	0.92	0.81 - 1.04	0.19
**Age**				1.01**	1.00 - 1.01	0.002
**Gender**				1.15***	1.06 - 1.24	< 0.001
**Race (ref = White)**						
**Black**				0.82**	0.71 - 0.94	0.005
**Hispanic**				1.01	0.85 - 1.19	0.92
**Other**				0.96	0.82 - 1.13	0.65
**Comorbidities**				0.91*	0.84 - 0.98	0.015
**Medication**				1.07	0.89 - 1.29	0.49
**CPD**				1.00	0.99 - 1.00	0.48
**Nicotine Dependence**				0.93***	0.91 - 0.95	< 0.001
**Years Smoked**				0.99***	0.99 - 1.00	< 0.001

© 2019 Cinciripini, PM, et al. *JAMA Network Open.*

eFigure 1. Longitudinal adjusted models of the impact of ever having cancer (CAHx+); having a smoking related (SMCA+) or unrelated (SMCa-) cancer; being a cancer survivor (S) or of individual cancer site on abstinence over time compared to never having cancer (CaHx-). Nominal p-values shown; multiple correction p-value for each cancer site vs. CaHx--=.006.

Cancer Characteristic	Relative Risk	95% CI	P Value
CaHx+	1.06	(0.95 - 1.18)	0.27
SMCa-	1.02	(0.90 - 1.14)	0.8
SMCa+	1.11	(0.99 - 1.25)	0.07
S	1.04	(0.89 - 1.20)	0.64
Breast	1.12	(0.96- 1.32)	0.15
Colorectal	0.99	(0.84 - 1.17)	0.89
Head & Neck	1.24	(1.08 - 1.42)	0.002
Lung	1.14	(0.99 - 1.32)	0.072
Lymphoma & Other Hem.	0.95	(0.82 - 1.12)	0.56
Melanoma & Other Skin	0.99	(0.81 - 1.2)	0.89
Other	1.03	(0.88 - 1.21)	0.71
Other GU	0.97	(0.82 - 1.15)	0.73
Prostate	0.94	(0.75 - 1.16)	0.55

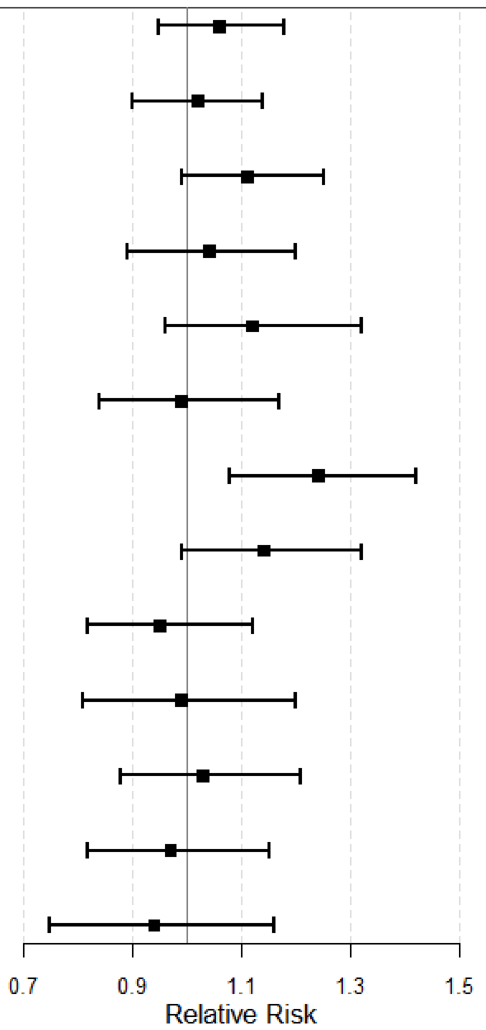

Reference Category: CaHx-

Other: Cervix, Soft Tissue, Brain & Other Nervous. System, Thyroid, Endocrine System, Eye & Orbit.**Other GU:** Uterus, Ovary, Breast Female, Other Female Genital Organs, Other Male Genital Organs, Urinary Bladder, Kidney & Renal Bladder.**Other GI:** Pancreas, Small Intestine, Stomach, Esophagus, Other Digestive Organs. **Other Hematologic**: Leukemia Acute, Multiple Myeloma, Other Hematologic System., Leukemia Chronic. **Other Skin**: Basal, Carcinoma NOS, Skin Trunk, Hemangiosarcoma.

© 2019 Cinciripini, PM, et al. *JAMA Network Open.*

eFigure 2. Adjusted Models of the Impact of Smoking Related Cancer and Survivor Groups on Abstinence Compared to No Cancer Group (CaHx-) by month and longitudinally for the multiply imputed sample. Nominal p values show. Adjusted p value for 2 comparisons (SMCa+/SMCa- vs. CaHx-) at 3 time points=.008; and for 1 comparions (S vs. CaHx-) at 3 time points= .016.

Cancer Characteristic	No. Abstinent/ No. Total	Abstinent (%)	Relative Risk	95% CI	P Value
CaHx-					
3 Months	234 / 540	43.3			
6 Months	218 / 496	43.9			
9 Months	187 / 454	41.2			
SMCa-					
3 Months	436 / 1019	42.8	0.99	(0.88 - 1.12)	0.89
6 Months	416 / 957	43.5	1	(0.88 - 1.14)	0.99
9 Months	385/ 900	42.3	1.07	(0.93 - 1.23)	0.36
Longitudinal			1.02	(0.90 - 1.14)	0.8
SMCa+					
3 Months	532 / 1146	46.4	1.09	(0.96 - 1.23)	0.17
6 Months	512 / 1075	47.6	1.09	(0.96 - 1.24)	0.16
9 Months	469 / 1019	46.1	1.15	(1.00 - 1.33)	0.055
Longitudinal			1.11	(0.99 - 1.25)	0.07
S					
3 Months	130 / 287	45.3	0.98	(0.82 - 1.17)	0.85
6 Months	129 / 274	47.1	1.06	(0.88 - 1.27)	0.54
9 Months	115 / 270	42.3	1.11	(0.91 - 1.36)	0.31
Longitudinal			1.04	(0.89 - 1.20)	0.64

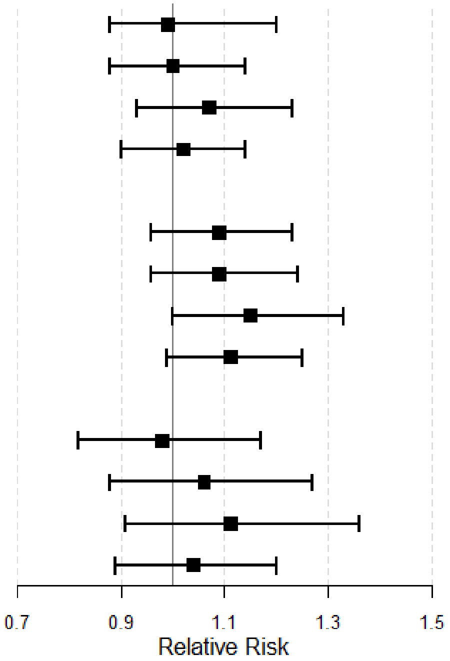

Reference Category: CaHx-

© 2019 Cinciripini, PM, et al. *JAMA Network Open.*

eReferences

1. Karam-Hage M, Oughli HA, Rabius V, et al. Tobacco Cessation Treatment Pathways for Patients With Cancer: 10 Years in the Making. *J Natl Compr Canc Netw*. 2016;14(11):1469-1477.
2. Miller WR, Rollnick S. *Motivational Interviewing: Preparing People to Change Addictive Behavior*. New York: Guilford Press; 1991.
3. Brown RA. Intensive behavioral treatment. In: Abrams DB, Niaura RS, Brown RA, Emmons KM, Goldstein MG, Moolchan ET, eds. *The tobacco dependence treatment handbook: A Guide to Best Practices*. New York: Guilford Press; 2003.
4. Fiore MC, Jaen CR, Baker TB, et al. Treating Tobacco Use and Dependence: 2008 Update, Clinical Practice Guideline. PM:18807274. Updated March 4, 2015.
5. Spitzer RL, Kroenke K, Williams JB. Validation and utility of a self-report version of PRIME-MD: the PHQ primary care study. Primary Care Evaluation of Mental Disorders. Patient Health Questionnaire. *JAMA*. 1999;282(18):1737-1744. doi:10.1001/jama.2012.8696.
6. Spitzer RL, Williams JBW, Kroenke K, et al. Utility of a new procedure for diagnosing mental disorders in primary care: The PRIME-MD 1000 study. *JAMA*. 1994;272:1749-1756. doi:10.1001/jama.282.18.1737.
7. Kroenke K, Spitzer RL, Williams JBW. The PHQ-15: Validity of a new measure for evaluating the severity of somatic symptoms. *Psychosom Med*. 2002;64(2):258-266.
8. Kroenke K, Spitzer RL, Williams JBW, Löwe B. The Patient Health Questionnaire Somatic, Anxiety, and Depressive Symptom Scales: A systematic review. *General Hospital Psychiatry*. 2010;32(4):345-359. doi:10.1016/j.genhosppsych.2010.03.006.
9. American Psychiatric Association. *Diagnostic and Statistical Manual of Mental Disorders. Fourth Edition*. Washington, D.C.: American Psychiatric Association; 1994.
10. Fagerström K. Determinants of tobacco use and renaming the FTND to the Fagerstrom Test for Cigarette Dependence. *Nicotine Tob Res*. 2012;14(1):75-78. doi:10.1093/ntr/ntr137.
11. Brown RA, Burgess ES, Sales SD, Whiteley JA, Evans DM, Miller IW. Reliability and validity of a smoking timeline follow-back interview. *Psychol Addict Behav*. 1998;12(2):101-112. doi:10.1037/0893-164X.12.2.101.
12. Rothman KJ, Greenland S, Lash TL. *Modern epidemiology.* Third edition. Philadelphia, Baltimore, New York: Wolters Kluwer Health Lippincott Williams & Wilkins; 2008.
13. Hymowitz N, Cummings KM, Hyland A, Lynn WR, Pechacek TF, Hartwell TD. Predictors of smoking cessation in a cohort of adult smokers followed for five years. *Tob Control*. 1997;6 Suppl 2:S57-62.
14. Guimond A-J, Croteau VA, Savard M-H, Bernard P, Ivers H, Savard J. Predictors of Smoking Cessation and Relapse in Cancer Patients and Effect on Psychological Variables: An 18-Month Observational Study. *Ann Behav Med*. 2017;51(1):117-127. doi:10.1007/s12160-016-9834-4.
15. Schnoll RA, Martinez E, Langer C, Miyamoto C, Leone F. Predictors of smoking cessation among cancer patients enrolled in a smoking cessation program. *Acta Oncol*. 2011;50(5):678-684.
16. Sachs DPL, Bostrom AG, Hansen MD. Predictors of smoking cessation success during nicotine patch therapy. *Ann Behav Med*. 1994;16(Suppl.)(Supplement (Proceedings from the Society of Behavioral Medicine Conference)):S42.
17. Greenland S. Model-based estimation of relative risks and other epidemiologic measures in studies of common outcomes and in case-control studies. *Am.J.Epidemiol.* 2004;160(4):301-305. doi:10.1093/aje/kwh221.
18. Zou GY, Donner A. Extension of the modified Poisson regression model to prospective studies with correlated binary data. *Statistical Methods in Medical Research*. 2013;22(6):661-670. doi:10.1177/0962280211427759.
19. Yelland LN, Salter AB, Ryan P. Performance of the modified Poisson regression approach for estimating relative risks from clustered prospective data. *American Journal of Epidemiology*. 2011;174(8):984-992. doi:10.1093/aje/kwr183.
20. Zou G. A Modified Poisson Regression Approach to Prospective Studies with Binary Data. *Am.J.Epidemiol.* 2004;159(7):702-706. doi:10.1093/aje/kwh090.
21. *Stata Statistical Software*. College Station, TX: StataCorp LP; 2017.
22. Greenland S. Interpretation and choice of effect measures in epidemiologic analyses. *Am.J.Epidemiol.* 1987;125(5):761-768. doi:10.1093/oxfordjournals.aje.a114593.
23. Greenland S, Holland P. Estimating standardized risk differences from odds ratios. *Biometrics*. 1991;47(1):319-322.
24. Sinclair JC, Bracken MB. Clinically useful measures of effect in binary analyses of randomized trials. *J Clin Epidemiol*. 1994;47(8):881-889.
25. Nurminen M. To use or not to use the odds ratio in epidemiologic analyses? *Eur J Epidemiol*. 1995;11(4):365-371.
26. Chen W, Qian L, Shi J, Franklin M. Comparing performance between log-binomial and robust Poisson regression models for estimating risk ratios under model misspecification. *BMC Med Res Methodol*. 2018;18(1):63. doi:10.1186/s12874-018-0519-5.

© 2019 Cinciripini, PM, et al. *JAMA Network Open.*

27. Lee KJ, Carlin JB. Multiple imputation for missing data: fully conditional specification versus multivariate normal imputation. *Am.J.Epidemiol.* 2010;171(5):624-632. doi:10.1093/aje/kwp425.
28. van Buuren S. *Flexible imputation of missing data*. Boca Raton, Fla.: CRC Press; 2012. Chapman & Hall/CRC interdisciplinary statistics series. http://search.ebscohost.com/login.aspx?direct=true&scope=site&db=nlebk&db=nlabk&AN=445864.
29. Hedeker D, Mermelstein RJ, Demirtas H. Analysis of binary outcomes with missing data: missing = smoking, last observation carried forward, and a little multiple imputation. *Addiction*. 2007;102(10):1564-1573. doi:10.1111/j.1360-0443.2007.01946.x.
30. Barnes SA, Larsen MD, Schroeder D, Hanson A, Decker PA. Missing data assumptions and methods in a smoking cessation study. *Addiction*. 2010;105(3):431-437. doi:10.1111/j.1360-0443.2009.02809.x.
31. Siddique J, Harel O, Crespi CM, Hedeker D. Binary variable multiple-model multiple imputation to address missing data mechanism uncertainty: application to a smoking cessation trial. *Statistics in medicine*. 2014;33(17):3013-3028. doi:10.1002/sim.6137.

© 2019 Cinciripini, PM, et al. *JAMA Network Open.*
